# Nutrient Limitation Mimics Artemisinin Tolerance in Malaria

**DOI:** 10.1128/mbio.00705-23

**Published:** 2023-04-25

**Authors:** Audrey C. Brown, Michelle D. Warthan, Anush Aryal, Shiwei Liu, Jennifer L. Guler

**Affiliations:** a Department of Biology, University of Virginia, Charlottesville, Virginia, USA; NIAID/NIH

**Keywords:** GSEA, RNA-seq, artemisinin, drug resistance evolution, nutrient starvation, parasitology, physiological media, stress response, tolerance

## Abstract

Mounting evidence demonstrates that nutritional environment can alter pathogen drug sensitivity. While the rich media used for *in vitro* culture contains supraphysiological nutrient concentrations, pathogens encounter a relatively restrictive environment *in vivo*. We assessed the effect of nutrient limitation on the protozoan parasite that causes malaria and demonstrated that short-term growth under physiologically relevant mild nutrient stress (or “metabolic priming”) triggers increased tolerance of a potent antimalarial drug. We observed beneficial effects using both short-term survival assays and longer-term proliferation studies, where metabolic priming increases parasite survival to a level previously defined as resistant (>1% survival). We performed these assessments by either decreasing single nutrients that have distinct roles in metabolism or using a media formulation that simulates the human plasma environment. We determined that priming-induced tolerance was restricted to parasites that had newly invaded the host red blood cell, but the effect was not dependent on genetic background. The molecular mechanisms of this intrinsic effect mimic aspects of genetic tolerance, including translational repression and protein export. This finding suggests that regardless of the impact on survival rates, environmental stress could stimulate changes that ultimately directly contribute to drug tolerance. Because metabolic stress is likely to occur more frequently *in vivo* compared to the stable *in vitro* environment, priming-induced drug tolerance has ramifications for how *in vitro* results translate to *in vivo* studies. Improving our understanding of how pathogens adjust their metabolism to impact survival of current and future drugs is an important avenue of research to slow the evolution of resistance.

## INTRODUCTION

*In vitro* culture provides microbes with a stable environment for growth with relatively little challenge to organismal homeostasis. For example, the supraphysiological concentrations of nutrients *in vitro* largely protect organisms from the stress of starvation when culturing best practices are followed. Yet, nutrient limitation occurs frequently *in vivo* (1 to 4). Therefore, the consequences of dynamic adaptation to stressors, such as nutrient variation, are largely missed in standard *in vitro* experiments.

**FIG 1:**
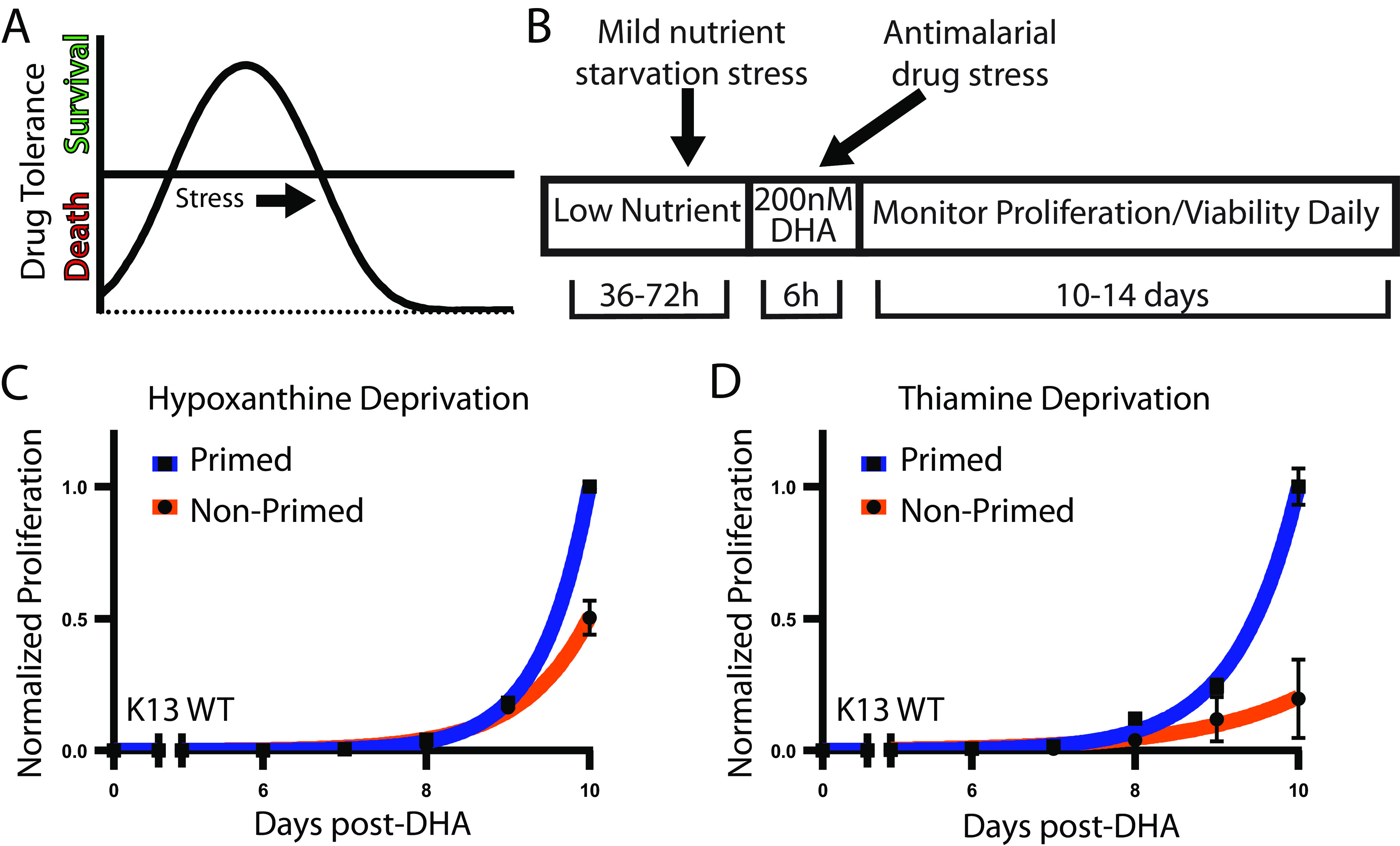
Mild nutrient stress prior to DHA treatment increases postdrug recovery. (A) The biphasic dose response to stress characteristic of hormesis. (B) Paradigm used for metabolic priming followed by DHA challenge and recovery. (C and D) Post-DHA recovery in standard media of P. falciparum primed in either low-hypoxanthine or thiamine-free media. Error bars represent SEM of technical replicates within one representative experiment. Results from all independent assays are detailed in Table S1 (low hypoxanthine, *n = *5; thiamine free, *n = *4).

Across the tree of life, the relationship between stress and fitness consequences is not necessarily linear. The adaptive benefits of low doses of mild environmental stress overwhelm the adverse effects from the stressor in a biphasic dose response phenomenon termed hormesis (5, 6) (Fig. 1A). Given this effect, adjustment of metabolism in response to mild nutrient stress has the potential to increase pathogen fitness and survival of subsequent stressors, such as drug pressure.

10.1128/mbio.00705-23.1 TABLE S1 Data for all recovery post-DHA or post-vehicle control experiments. Download Table S1, PDF file, 0.5 MB. Copyright © 2023 Brown et al. 2023 Brown et al. https://creativecommons.org/licenses/by/4.0/ This content is distributed under the terms of the Creative Commons Attribution 4.0 International license.

Indeed, metabolic adjustments under varied nutritional environments alter drug sensitivity across diverse pathogens (7, 8). In *Trypanasoma cruzi*, reduction of exogenous glutamine decreases flux through the sterol synthesis pathway, rendering parasites sensitive to azole drugs (9). Pseudomonas aeruginosa supplementation with specific carbon sources increases susceptibility to tobramycin (10). Increasing proline concentration confers halofuginone resistance in Plasmodium falciparum (11). The nutritional environment *in vitro* is highly divergent from conditions that pathogens are exposed to in the context of human infection, with nutrient levels frequently found at considerably higher levels in culture (12 to 14). Discrepancies between *in vitro* and *in vivo* environments can lead to reduced translatability of findings from nonphysiological culture-based experiments when metabolic flux through pathways is altered (15, 16). For example, glycerol metabolism sensitizes Mycobacterium tuberculosis to pyrimidine-imidazoles through accumulation of toxic metabolites; however, these drugs are ineffective *in vivo*, where changes in nutrient availability lead to differences in carbon metabolism (15).

These previously characterized changes in pathogen drug sensitivity as a consequence of varied nutritional environment typically depend on either (i) changes in flux through a specific metabolic pathway relevant to a given drug target or (ii) a general slowing of growth leading to decreased uptake and activation of drug. Here, we present a case where nutrient stress induces a change in drug sensitivity of the human malaria parasite, P. falciparum. We demonstrate that short-term growth under mild, physiologically relevant nutrient limitation leads to superior recovery from drug treatment. We observe this effect under distinct conditions that impact parasites differently; therefore, it is not dependent on a change in flux within a specific metabolic pathway nor an overall decrease in growth rate (as in the above examples). Importantly, the increased survival rates achieve a level that reaches the field’s definition of “resistance,” yet genetic mutations that confer resistance are not necessary. Finally, we identify prosurvival pathways that are triggered by nutrient limitation to prime the parasite to better withstand drug treatment. This study emphasizes the links among nutrient limitation, prosurvival pathways, and sensitivity to drugs and may hold important implications for the evolution of drug-resistant pathogens.

## RESULTS

### Metabolic priming and recovery from DHA.

We sought to determine the effect of mild nutrient stress on the survival of P. falciparum. Asynchronous parasite cultures were subjected to short-term (36 to 72 h) incubation in media restricted of either hypoxanthine or thiamine (termed “metabolic priming”; Table 1). The duration of incubation and level of nutrient limitation for these metabolites were optimized to induce mild stress while keeping the majority of the parasite population viable (Fig. S1).

**TABLE 1:** Length and concentration of nutrient limitation for metabolic priming

Nutrient restricted	Class of restricted nutrient	Conc. of restricted nutrient	Length of restriction
Hypoxanthine^*a*^	Purine	0.5 uM	48 to 72 h
Thiamine^*b*^	Cofactor	0.0 uM	36 h
Multiple (HPLM)^*b*^	Multiple	Variable	≥7 days

a Priming was considered successful for this nutrient when an approximately 10 to 50% growth decrease occurred, there was little to no decrease in mitochondrial membrane potential (MMP) (approximately <10% drop), and ring stage percentages were within <10% ± nonprimed.

b Priming was considered successful for these nutrients when the latter two above conditions (MMP and staging) were met but no growth reduction criteria were required.

10.1128/mbio.00705-23.4 FIG S1 Metabolic priming does not drastically impact parasite viability. Successful low nutrient metabolic priming leads to very small decreases in viability compared to standard media, nonprimed controls. The percentage of parasites (SYBR Green I cells) also positive for MitoProbe DiIC1(5) staining (an indicator of mitochondrial membrane potential; MMP) was used as a proxy for determining viability. *n = *3 to 10 per condition. Bars represent SEM. Download FIG S1, PDF file, 0.5 MB. Copyright © 2023 Brown et al. 2023 Brown et al. https://creativecommons.org/licenses/by/4.0/ This content is distributed under the terms of the Creative Commons Attribution 4.0 International license.

We treated parasites grown in either metabolic priming conditions or standard media (formulations detailed in Materials and Methods) with a rapid-acting, highly potent antimalarial drug (200 nM dihydroartemisinin [DHA] for 6 h, Fig. 1B). Following removal of drug and continued culture in standard media, primed parasites showed a significant increase in cumulative proliferation compared to nonprimed controls (low hypoxanthine mean day 10 difference: 130%, *n* = 5, *P = *0.0126; thiamine-free: 173%, *n = 4*, *P = *0.0074) (Fig. 1C and D). We detected increased parasite recovery from DHA in both priming conditions despite the restricted nutrients under each condition being of unrelated metabolite classes and irrespective of the parasite line (*Dd2* originally from Southeast Asia and *NF54* from Africa; Table S1). The increased cumulative proliferation is driven by altered response to DHA treatment; we only observed this effect in primed parasites that were subjected to DHA treatment but not in those treated with vehicle alone (Fig. S2).

10.1128/mbio.00705-23.5 FIG S2 Metabolic priming does not impact post-DHA recovery without drug pulse. (A and B) Growth in standard media following metabolic priming under (A) low hypoxanthine or (B) thiamine-free conditions (Table 1) without a DHA drug pulse (vehicle is DMSO). Bars represent SEM of technical replicates within one representative experiment. Results from independent assays are detailed in Table S1 (*n = *2 per condition). Download FIG S2, PDF file, 0.5 MB. Copyright © 2023 Brown et al. 2023 Brown et al. https://creativecommons.org/licenses/by/4.0/ This content is distributed under the terms of the Creative Commons Attribution 4.0 International license.

During metabolic priming experiments, the level of stress applied to the parasites impacted whether we observed superior recovery. A >10% growth reduction over the course of low hypoxanthine metabolic priming was found to facilitate the phenotype (Fig. 1C), while failure to detect a growth phenotype (Fig. S3A) or overly stressful conditions (Fig. S3B) were detrimental to post-DHA recovery. These data support a biphasic dose response to metabolic priming characteristic of hormesis. However, this pattern is dependent on the limiting nutrient; in other conditions, growth during priming did not serve as a proxy for estimating dose response. Thiamine-free priming did not lead to growth reduction but still led to increased cumulative proliferation following DHA treatment (Table S1, Fig. 1D). The differential effect of priming treatments on growth is likely due to parasite ability to scavenge or *de novo* synthesize thiamine (17), whereas P. falciparum are strictly purine auxotrophs.

10.1128/mbio.00705-23.6 FIG S3 Increased post-DHA recovery requires a specific range of stress during metabolic priming. (A and B) Post-DHA recovery in standard media following low hypoxanthine metabolic priming where priming led to (A) less than 10% growth reduction or (B) greater than ~60% growth reduction and >10% reduction in MMP compared to nonprimed controls. Bars represent SEM of technical replicates within one independent experiment. Download FIG S3, PDF file, 0.4 MB. Copyright © 2023 Brown et al. 2023 Brown et al. https://creativecommons.org/licenses/by/4.0/ This content is distributed under the terms of the Creative Commons Attribution 4.0 International license.

### Impact of metabolic priming on parasite stage and DHA survival rates.

**FIG 2:**
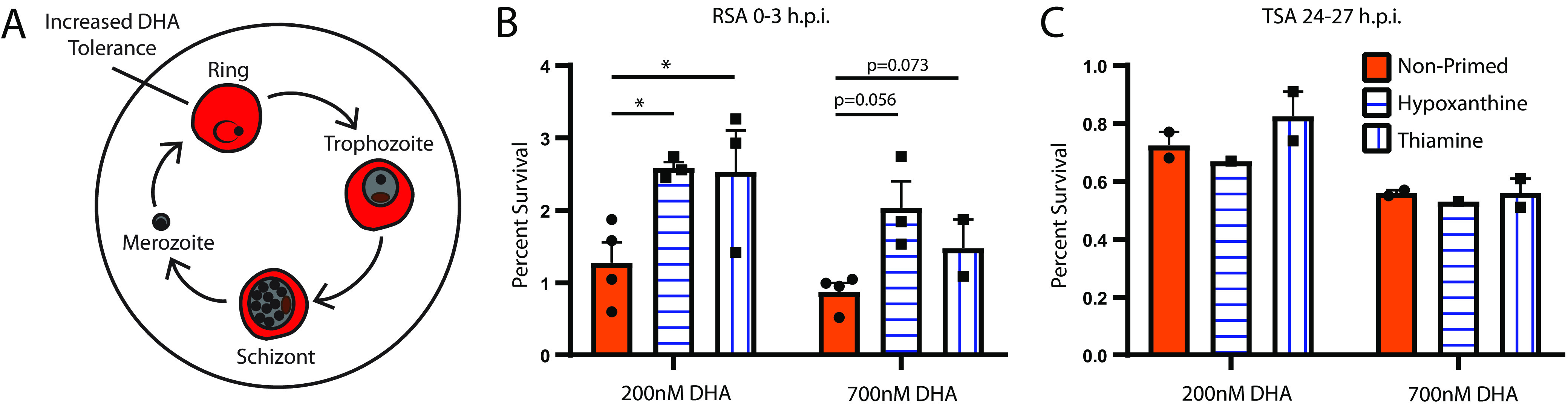
Increases in post-DHA survival are mediated by ring stage parasites. (A) P. falciparum asexual blood-stage replication cycle. DHA tolerance is highest in ring stage parasites 0 to 3 h postinvasion (18). (B and C) Percent survival for early rings (B; 0 to 3 h postinvasion, *n* = 3 to 4) or trophozoites (C; 24 to 27 h postinvasion, *n = *1 to 2) measured 66 h after treatment with 200 or 700 nM DHA for 6 h. Bars represent SEM of independent experiments. Parasite line: *Dd2*. RSA *P* values are as follows: nonprimed versus low hypoxanthine: 200 nM = 0.031, 700 nM = 0.056; nonprimed versus thiamine free: 200 nM = 0.038, 700 nM = 0.073. TSA *P* values are as follows: nonprimed versus low hypoxanthine: 200 nM = 0.407, 700 nM = 0.930; nonprimed versus thiamine free: 200 nM = 0.796, 700 nM = 0.999. *P* values of <0.05 are indicated with an asterisk (*).

DHA survival patterns are dependent on the parasite life cycle stage (with highest survival at 0 to 3 h postinvasion [18]; Fig. 2A). Therefore, we evaluated (i) the stage effects of metabolic priming and (ii) the stage specificity of the proliferation effect.

First, we compared parasite stage distribution in primed and nonprimed populations to assess if subtle changes in life cycle stage occur during priming conditions (i.e., stage effects). We did not detect any significant differences in the percentage of ring stage parasites in nonprimed compared to primed samples using microscopy and flow cytometry (nonprimed mean ± SEM [standard error of the mean]: 61.6% ± 2.1%; primed: 61.3% ± 2.3%; *P = *0.67) (Table S1; Fig. S4A and B). To further assess parasite cell cycle progression, we assessed stage composition from primed and nonprimed RNA expression profiles. Staging across groups was largely consistent except for a higher-than-expected schizont population in hypoxanthine primed cultures (Fig. S4C). This population most likely represents uninvaded merozoites, as decreased reinvasion is the presumed mechanism for the reduced parasitemia seen in hypoxanthine-primed cultures. Uninvaded merozoites are not included in the flow cytometry gating strategy, used to determine starting parasitemia for growth curve normalization. Thus, we removed the schizont population from CIBERSORTx results before reassessing population staging distribution. The relative proportions of rings, early trophozoites, and late trophozoites were similar across groups (Fig. S4D). For each stage, nonprimed (RPMI) samples closely aligned with the average across all samples (Fig. S4E), indicating primed samples are not skewed toward more DHA-tolerant ring stages compared to nonprimed samples.

10.1128/mbio.00705-23.7 FIG S4 Priming does not affect parasite stage makeup. (A) Micrographs of synchronous ring stage parasites after priming (magnification = 100×). (B) Flow cytometry plots of synchronous nonprimed and primed infected red blood cells. Plots are partitioned into single nuclei (early-stage parasites) and multiple nuclei (late-stage parasites) determined by SYBR Green I DNA staining intensity. (C and D) Distribution of parasite stages computationally inferred from RNA expression profiles before (C) and after (D) removal of the schizont/uninvaded merozoite fraction. (E) Distribution of inferred staging from panel D visualized across all treatment groups. Download FIG S4, PDF file, 0.6 MB. Copyright © 2023 Brown et al. 2023 Brown et al. https://creativecommons.org/licenses/by/4.0/ This content is distributed under the terms of the Creative Commons Attribution 4.0 International license.

Second, we performed shorter-term DHA survival assays on highly synchronous parasite populations following priming to determine if changes in parasite recovery are stage specific. To assess the behavior of early-stage parasites (Fig. 2A), we performed “ring stage survival assays” (RSAs) on highly synchronous populations of 0- to 3-h rings (18). Metabolic priming prior to DHA treatment increased survival of ring stage parasites (Fig. 2B). We observed a mean 2.1-fold increase for both 200-nM and 700-nM DHA, which are the concentrations used for our study (Fig. 1C and D) and the standard concentration for DHA RSAs, respectively. Notably, metabolic priming increased survival percentages to a level that is considered resistant (>1% for 700-nM DHA for 6 h [18]). Additionally, we tested later-stage parasites (24 to 27 h) in a modified RSA that assesses trophozoite survival (TSA [18]; Fig. 2C). We detected no significant increase in DHA survival following metabolic priming, indicating the benefit of metabolic priming is ablated in trophozoites (Fig. 2D). In addition to showing ring stage specificity of the effect, these experiments also showed that higher cumulative proliferation in primed samples post-DHA is a result of an increased percentage of parasites surviving DHA treatment, as opposed to increased proliferation per parasite.

### Metabolic priming of parasites with genetic DHA resistance.

**FIG 3:**
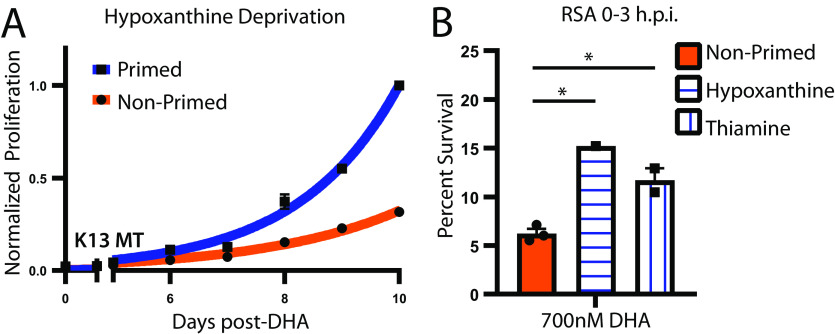
Metabolic priming effects are conserved in parasites carrying genetic DHA resistance. (A) Post-DHA recovery of DHA-tolerant parasite line in standard media and primed in low-hypoxanthine media. Bars represent SEM of technical replicates within one representative experiment. (B) Percent survival for early rings (0 to 3 h postinvasion) measured 66 h after treatment with 700 nM DHA for 6 h (*n *= 1 to 3). Bars represent SEM of independent experiments. Parasite line: *MRA-1238*. RSA *P* values are as follows: nonprimed versus low hypoxanthine: 700 nM = 0.012; nonprimed versus thiamine free: 700 nM = 0.026. *P* values of <0.05 are indicated with an asterisk (*).

We sought to assess if the survival benefits of metabolic priming also apply to genetically DHA-resistant parasites (RSA >1% [18]). Using our long-term growth assay, we found that metabolic priming does improve postdrug recovery of a moderately DHA-resistant parasite strain (Fig. 3A; MRA-1238, RSA: 6.2% survival, *kelch13* I543T mutant [19]). We confirmed this effect using RSAs; metabolic priming increases survival of a high DHA concentration (700 mM) by a mean of 2.1-fold (Fig. 3B). This parasite line was not tested at the lower 200-nM concentration due to its known resistance status; other DHA-resistant lines (i.e., MRA 1240, RDA: 88.2% survival, *kelch 13* R539T) could not be assessed because of their high tolerance to 700-nM DHA levels. These data in combination with data generated using DHA-sensitive lines (Fig. 1 and 2, Table S1; *Dd2* and *NF54*), indicate that increased DHA survival following metabolic priming triggers an intrinsic response that is not dependent on genetic background.

### Contribution of autophagy to metabolic priming effects.

When nutrients are limiting, autophagy is stimulated to recycle endogenous components for critical pathways. To explore the role of autophagy in metabolic priming, we used a parasite strain with a conditional regulation of ATG8 expression; removal of the small molecule, anhydrotetracycline (aTc), leads to knockdown of ATG8, which is essential for autophagosome formation and therefore essential for successful macroautophagy (20). When we compared post-DHA recovery between parasites with low versus normal levels of ATG8 expression (Fig. S5A and B), all metabolically primed samples displayed increased survival relative to nonprimed samples regardless of ATG8 status (Fig. S5C and D, Table S1). Given that autophagy plays a direct role in the recovery of DHA treatment (21, 22) and that we observed a 4-day difference of postdrug recovery between nonprimed parasites with normal and low ATG8 expression (time to 5% parasitemia, ATG8 On: 7.3 days ± 0.7 days [SEM], ATG8 Off: 11.5 days ± 0.5 days; Fig. S5E), we were not able to conduct RSAs with these parasite lines (requires measurement at ~72 h post-DHA [18]).

10.1128/mbio.00705-23.8 FIG S5 Priming improves post-DHA survival regardless of autophagy status. (A and B) Effect of knockdown on ATG8 protein levels in ATG8 TetR-Dozi parasites. Bars represent SEM (*n = *3). (C and D) Post-DHA recovery in two independent biological experiments of hypoxanthine-primed ATG8 TetR-Dozi parasites with ATG8 expression on (C) or off (D). Bars represent SEM of technical replicates within one independent experiment. (E) Growth recovery of nonprimed parasites differing in ATG8 expression status (*n = *2). aTc, anhydrotetracycline, small molecule that allows induction of ATG8 expression; IPP, isoprenoids, necessary metabolite for P. falciparum survival in the absence of ATG8. Download FIG S5, PDF file, 0.6 MB. Copyright © 2023 Brown et al. 2023 Brown et al. https://creativecommons.org/licenses/by/4.0/ This content is distributed under the terms of the Creative Commons Attribution 4.0 International license.

### Impact of physiological growth conditions on DHA survival.

**FIG 4:**
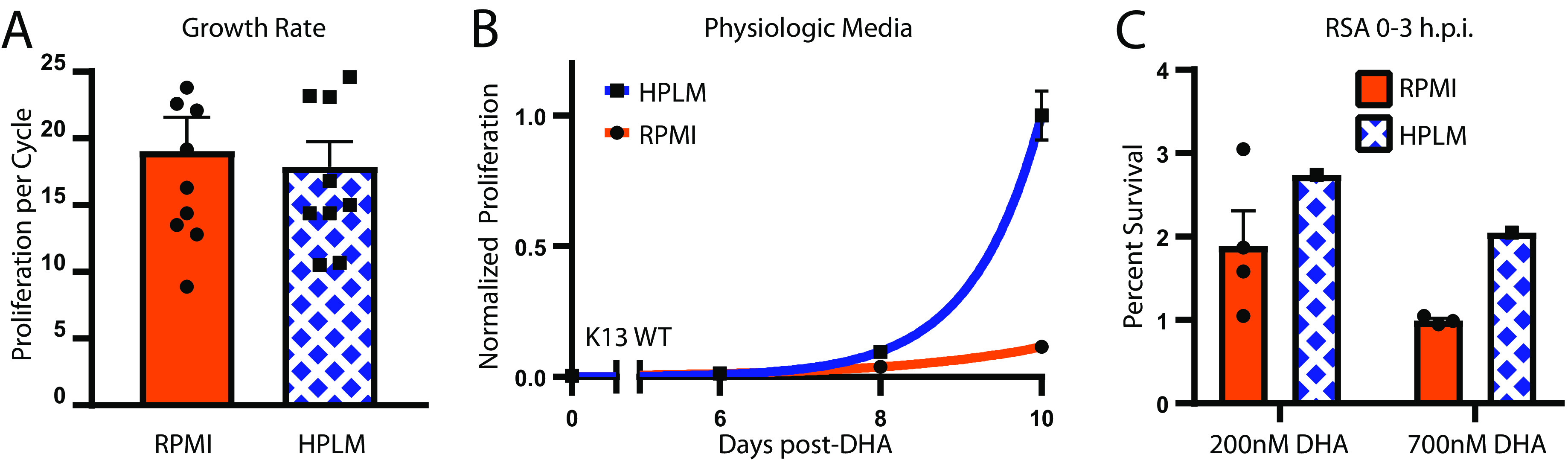
Physiologic media increases post-DHA recovery. (A) Average growth rate of *Dd2* parasites propagated in RPMI- versus HPLM-based media. *n = *10 representing 5 replication cycles of two independent growth assays. (B) Post-DHA recovery of P. falciparum propagated in RPMI- or HPLM-based media. Parasites were adapted to HPLM-based media at least 7 days prior to drug treatment and maintained in this media for 10 days of growth evaluation. Bars represent SEM of technical replicates within one representative experiment. Results from independent assays are detailed in Table S1 (*n = *2). (C) Percent survival for early rings (0 to 3 h postinvasion) measured 66 h after treatment with 200 or 700 nM DHA for 6 h (*n *= 1). Parasites were adapted to HPLM-based media at least 7 days prior to drug treatment and maintained in this media through the RSA reading. Bars represent SEM of independent experiments. Parasite line: *Dd2*. RPMI, Roswell Park Memorial Institute Media; HPLM, human plasma-like media.

Levels of many nutrients are dramatically lower *in vivo* compared to the supraphysiological levels in standard RPMI-based media formulations (12 to 14). We hypothesized that the nutritional differences between RPMI and human plasma are sufficient to induce a metabolic priming-like effect similar to that seen with our single nutrient depletion conditions. To test this, we utilized human plasma-like media (HPLM) as a base for P. falciparum culture (23). Under routine propagation conditions (*see* Materials and Methods), parasites maintained in HPLM-based media grew equivalent to those in RPMI-based media (HPLM [mean ± SEM]: 18 ± 1.9-fold per replication cycle; RPMI: 19 ± 2.5-fold; Fig. 4A).

Propagation in HPLM-based media for at least 7 days prior to DHA pulse led to superior postdrug recovery compared with parasites in RPMI-based media (mean day 10 difference: 459%, *n* = 2, *P = *0.0037; Fig. 4B, Table S1). As with low-hypoxanthine and thiamine-free priming conditions, growth in HPLM also increased DHA survival in RSAs on highly synchronized parasites (Fig. 4C, mean difference of 1.4-fold, including both 200 and 700 nM DHA samples). Once again, we did not detect significant differences in parasite staging in primed parasites using microscopy, flow cytometry, and RNA expression profiles (Fig. S4A, B, D, and E). These data provide evidence that related prosurvival changes are occurring in response to both single nutrient deficiency and broader reductions of environmental nutrients.

### Parasite transcriptome after metabolic priming.

**FIG 5:**
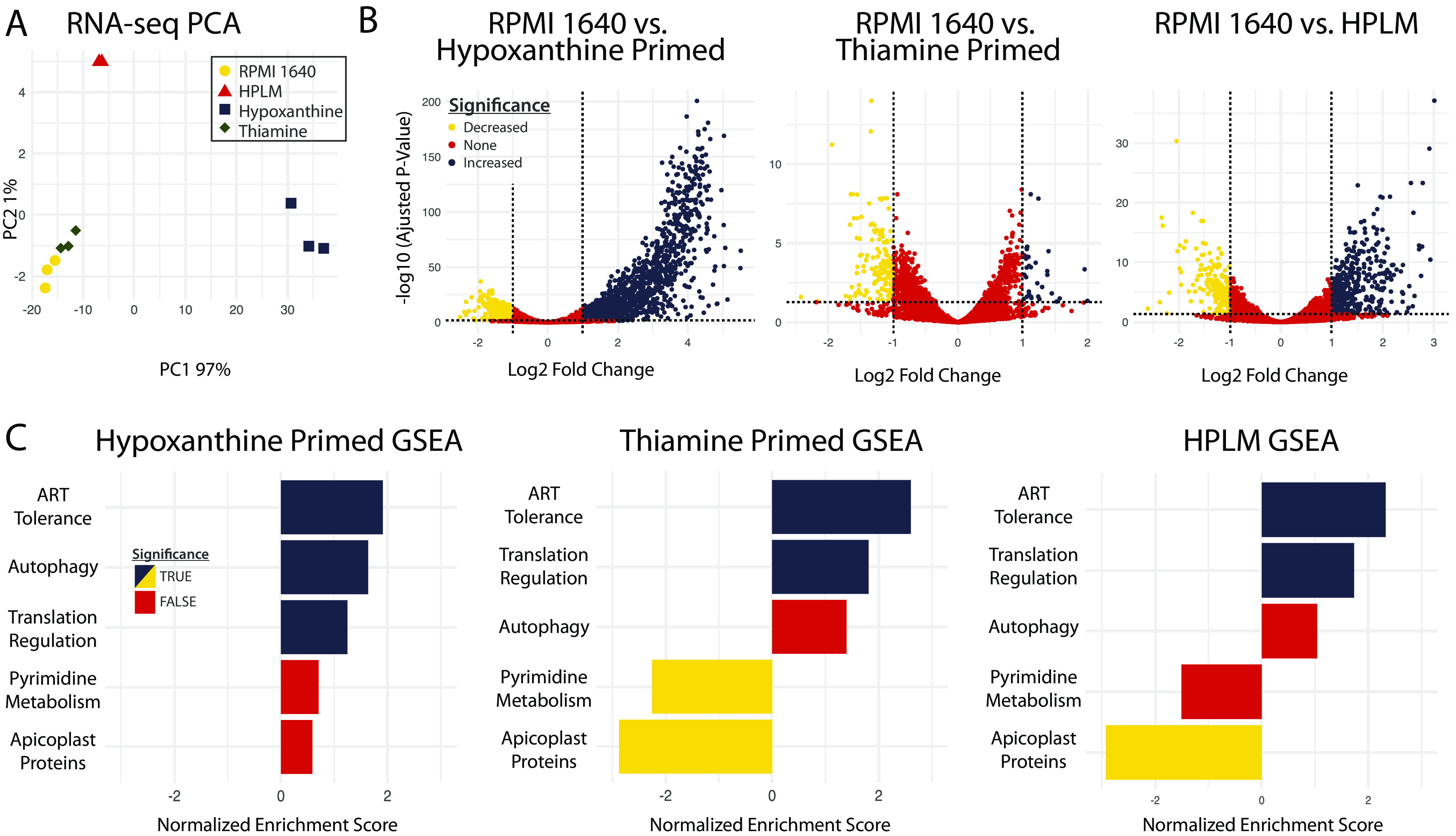
Metabolic priming induces artemisinin tolerance-like transcriptional changes. (A) PCA plot of gene expression from each condition. (B) Volcano plots of differentially expressed genes for each condition. Log_2_-fold change thresholds set at −1 and +1. Adjusted *P* value set to ≤0.05. (C) GSEA results for selected pathways. TRUE and FALSE = adjusted *P* value of <0.05 and >0.05, respectively. RPMI, Roswell Park Memorial Institute Media; HPLM, human plasma-like media; GSEA, gene set enrichment analysis; PCA, principal-component analysis.

To more fully explore pathways that are altered in response to nutritional environment, we assessed gene expression in synchronous ring stage nonprimed parasites and parasites primed with low hypoxanthine, thiamine free, or with HPLM conditions prior to DHA treatment. Ten-day post-DHA survival confirmed that hypoxanthine- and thiamine-free priming increased post-DHA survival in the RNA-seq samples (Table S1). Principal-component analysis showed that each group had a distinct transcriptional profile, with low hypoxanthine primed parasites clustering separate from the other three groups on PC1 and the remaining groups separating on PC2 (Fig. 5A). This pattern parallels the number of significantly deferentially expressed (DE) genes detected in experimental groups compared to nonprimed control samples, with low hypoxanthine primed parasites yielding the largest number of differences (Fig. 5B).

To identify prosurvival pathways, we performed gene set enrichment analysis (GSEA) across priming conditions (Fig. 5C). Some metabolic pathways not previously implicated in stress responses, but important for parasite biology, were altered in a condition-dependent manner. For example, pyrimidine biosynthesis and apicoplast metabolism were significantly downregulated in thiamine-free and HPLM groups, respectively. Similarly, autophagy was only significantly upregulated in low hypoxanthine conditions (Fig. 5C). This result, combined with our observation that metabolic priming remains effective in autophagy-depleted parasites (Fig. S5C and D), indicates that autophagy is unlikely to be a primary driver of the observed phenotype.

Overall, GSEA showed that 25 of 463 total pathways were significantly enriched across all groups (Table S2), including pathways heavy in exported proteins (e.g., protein–protein interactions between P. falciparum and the red blood cell). Upregulation of exported proteins has been previously observed in both the artemisinin resistance transcriptome and as a response to nutrient limitation (24 to 26). Translational regulation was also significantly upregulated in all three conditions in concordance with previous knowledge of the eIF2α-mediated response under nutrient stress (Fig. 5C, Table S2). This pathway includes genes with roles in processes known to impact DHA survival, including the unfolded protein response (UPR) and proteasomal degradation (Table S2).

10.1128/mbio.00705-23.2 TABLE S2 Gene set enrichment analysis results. Download Table S2, XLSX file, 0.3 MB. Copyright © 2023 Brown et al. 2023 Brown et al. https://creativecommons.org/licenses/by/4.0/ This content is distributed under the terms of the Creative Commons Attribution 4.0 International license.

Due to the large number of DE genes following hypoxanthine priming, and relatively small number in thiamine-free samples, it was necessary to limit the complexity of our results to further assess individual transcripts contributing to our phenotype. We specifically focused on the top 15 up- and downregulated genes in each group as determined by lowest adjusted *P* values. This yielded 37 up- and 42 downregulated unique genes across the three treatment conditions as there was some overlap between groups. From this list of genes, we identified those that had an adjusted *P* value of ≤0.05 in all groups as a curated list of 26 up- and 20 downregulated genes of interest (Table 2; Fig. S6). This curated list contained many genes implicated in artemisinin genetic tolerance, such as *coronin* (PF3D7_1251200), a gene known to be sufficient to induce artemisinin tolerance when mutated (27). Compared to a list of 181 genes known to be basally altered (without active DHA treatment; Table S3) in genetically artemisinin-tolerant parasites (27 to 32), 8 of our curated genes were represented (Table 2). Further, changes in this curated set of 181 genes (designated “ART Tolerance” in Fig. 5C) were highly enriched by GSEA in all groups (in the top 2% of 463 pathways by adjusted *P* value). These data indicate that nutrient limitation remodels metabolism in parasites to induce an artemisinin tolerance-like state.

**TABLE 2:** Genes of interest curated from differentially expressed gene lists

Gene ID^*a*^	Product description
Upregulated	
PF3D7_0214600*	serine/threonine protein kinase STK2, putative
PF3D7_0402200	surface-associated interspersed protein 4.1 (SURFIN 4.1), pseudogene
PF3D7_0418600	regulator of chromosome condensation, putative
PF3D7_0613900	myosin E, putative
PF3D7_0618000	conserved *Plasmodium* membrane protein, unknown function
PF3D7_0707300	rhoptry-associated membrane antigen
PF3D7_0724900	kinesin-20, putative
PF3D7_0817600*	conserved protein, unknown function
PF3D7_0822900*	PhIL1-interacting candidate PIC2
PF3D7_0903600	conserved protein, unknown function
PF3D7_0911100	START domain-containing protein, putative
PF3D7_0919900	regulator of chromosome condensation-PP1-interacting protein
PF3D7_1026600	conserved *Plasmodium* protein, unknown function
PF3D7_1035300*	glutamate-rich protein GLURP
PF3D7_1126700	conserved *Plasmodium* protein, unknown function
PF3D7_1143100	AP2 domain transcription factor AP2-O
PF3D7_1145200*	serine/threonine protein kinase, putative
PF3D7_1206300*	conserved *Plasmodium* protein, unknown function
PF3D7_1223100*	cAMP-dependent protein kinase regulatory subunit
PF3D7_1251200*	coronin
PF3D7_1252400	reticulocyte binding protein homologue 3, pseudogene
PF3D7_1327300	conserved *Plasmodium* protein, unknown function
PF3D7_1335400	reticulocyte binding protein 2 homologue a
PF3D7_1356800	serine/threonine protein kinase ARK3, putative
PF3D7_1371600	erythrocyte binding like protein 1, pseudogene
PF3D7_1423300	serine/threonine protein phosphatase 7
Downregulated	
PF3D7_0308000	DNA polymerase delta small subunit, putative
PF3D7_0510100	KH domain-containing protein, putative
PF3D7_0512800	conserved *Plasmodium* protein, unknown function
PF3D7_0711000	AAA family ATPase, CDC48 subfamily
PF3D7_0715900	cation diffusion facilitator family protein, putative
PF3D7_0801100	28S ribosomal RNA
PF3D7_0802200	1-cys peroxiredoxin
PF3D7_0923800	thioredoxin reductase
PF3D7_1130900	conserved *Plasmodium* protein, unknown function
PF3D7_1239200	AP2 domain transcription factor, putative
PF3D7_1303800	conserved *Plasmodium* protein, unknown function
PF3D7_1322300	BRCT domain-containing protein, putative
PF3D7_1330600	elongation factor Tu, putative
PF3D7_1334500	MSP7-like protein
PF3D7_1359600	conserved *Plasmodium* protein, unknown function
PF3D7_1371800	*Plasmodium* exported protein, unknown function
PF3D7_1419200	thioredoxin-like protein, putative
PF3D7_1440200	stromal-processing peptidase, putative
PF3D7_1443900	heat shock protein 90, putative
PF3D7_MIT04100	large subunit ribosomal RNA fragment D

a Asterisk indicates genes found to be basally altered in artemisinin mutants (from Table S3).

10.1128/mbio.00705-23.3 TABLE S3 Studies measuring basal differences between artemisinin-resistant and -sensitive parasites using omics approaches. Download Table S3, PDF file, 0.6 MB. Copyright © 2023 Brown et al. 2023 Brown et al. https://creativecommons.org/licenses/by/4.0/ This content is distributed under the terms of the Creative Commons Attribution 4.0 International license.

10.1128/mbio.00705-23.9 FIG S6 Clustering on DEGs reveals differences between nonprimed and treatment group samples but with low intragroup variability. Hierarchical clustering of Pearson’s correlation coefficients utilizing counts of genes listed in Table 2. ALB, nonprimed; Hx, hypoxanthine primed; HPLM, human plasma-like media; TF, thiamine-free primed. Download FIG S6, PDF file, 0.4 MB. Copyright © 2023 Brown et al. 2023 Brown et al. https://creativecommons.org/licenses/by/4.0/ This content is distributed under the terms of the Creative Commons Attribution 4.0 International license.

## DISCUSSION

**FIG 6:**
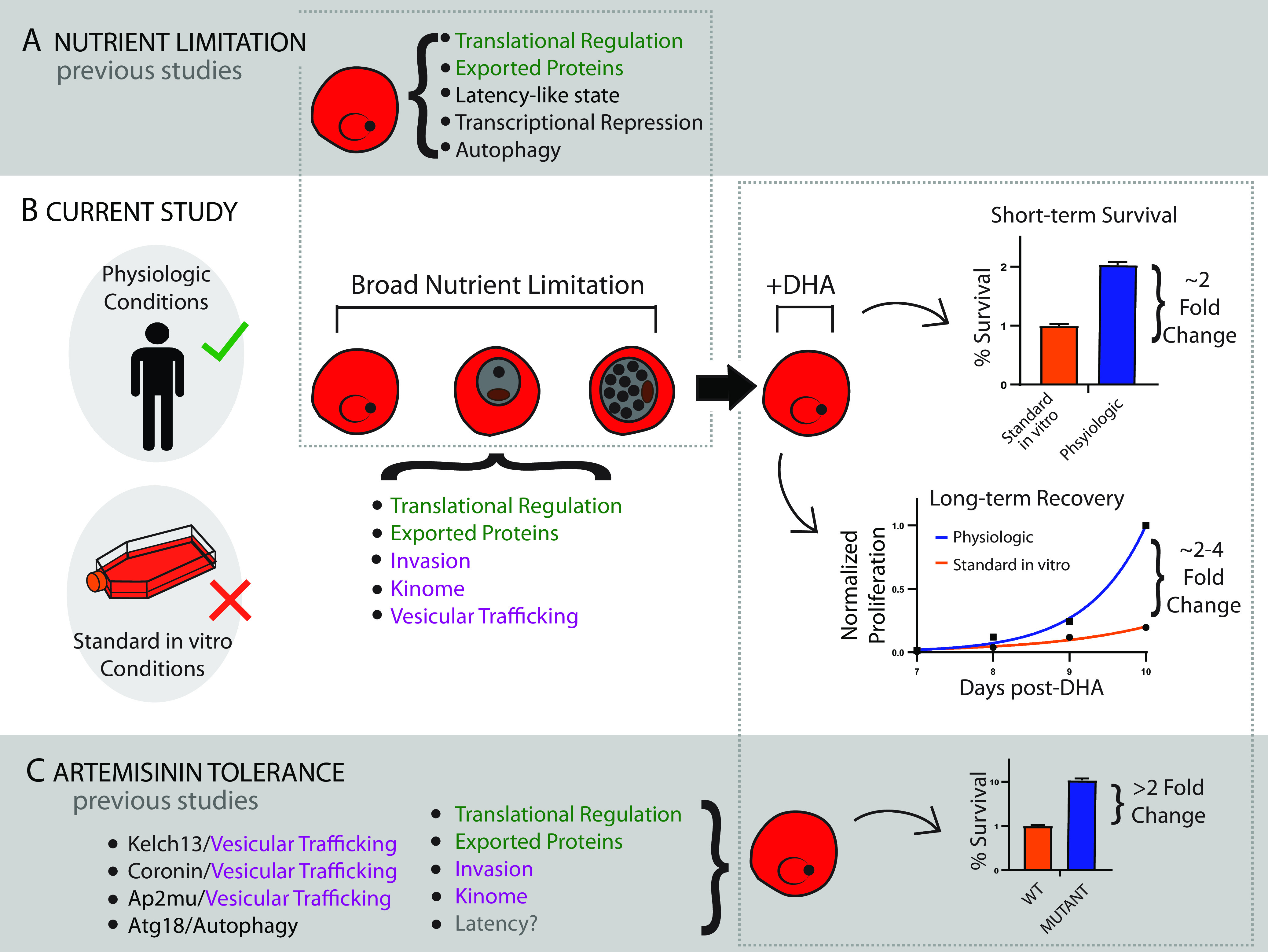
Integration of information related to nutrient deprivation and artemisinin tolerance. (A) Previous studies identified that nutrient limitation has metabolic impacts on *Plasmodium* parasites. Translational regulation: eIF2α (22, 35); exported proteins (25); latency-like state (33); transcriptional repression (34); autophagy: ATG5/8 (61). (B) The current studies have identified a direct link between nutrient limitation and parasite survival following dihydroartemisinin (DHA) treatment. These conditions are more relevant in the human host than in standard *in vitro* conditions. Transcriptional changes induced by nutrient limitation mimic those that contribute to artemisinin tolerance. Translational regulation: Fig. 5C, Table S2, “Transl_Regul” (285 genes); exported proteins: Table S2 “Meroz_RBClig” (19 genes) and “DomSurfaceProt” (14 genes); invasion: Table 2 (i.e., GLURP, Phil1), Table S2 “Erythrocyteinvasionpath” (8 genes), “Merozoiteproteins” (89 genes); Kinome: Table 2 (i.e., STK2, STK, CDPK, ARK3), Table S2 “Phospho_mero” (482 genes); vesicular trafficking: Table 2 (i.e., coronin). (C) Previous studies identified pathways associated with artemisinin tolerance. Vesicular trafficking: kelch13 (40), coronin (27), Ap2μ (62); autophagy: ATG18 (21, 22); translational regulation: unfolded protein response (63), proteasomal degradation (UBP1 [62]); exported proteins (24, 26); invasion: GLURP and Phil1 (28); kinome: STK2, STK, CDPK (28); latency: gray text, unclear if distinct from nutrient-dependent latency or directly contributes to tolerance (37, 64).

In the current study, we have demonstrated that physiologically relevant nutrient limitation stimulates intrinsic prosurvival pathways in P. falciparum (Fig. 1 and 4). Our analyses indicate genes within these altered pathways directly contribute to genetic artemisinin tolerance (Fig. 5C); thus, we speculate that nutrient limitation offers a preamble for resistance evolution. Our findings connect to other findings on cellular responses to both nutrient limitation and tolerance (Fig. 6) and provide a framework for how the nutritional environment impacts pathogen homeostasis. We explore these connections and their relevance below.

### Limiting nutrient levels for malaria parasites *in vitro*.

The supraphysiological concentrations of nutrients found in common media formulations, such as RPMI 1640, can potentiate genetic, transcriptional, and metabolic changes in parasite biology (reviewed in references 12 and 14). Here, we observed superior parasite recovery following limitation of single nutrients in *in vitro* growth media; hypoxanthine and thiamine were chosen in part due to contrasting metabolic attributes and due to potential relevance to conditions P. falciparum may experience *in vivo* (see further discussion below). *Plasmodium* lacks the ability to *de novo* synthesize purines, instead requiring scavenging from the environment. In serum-free malaria cultures, the purine derivative, hypoxanthine, is a standard additive to fulfill this requirement. In contrast, *Plasmodium* can *de novo* both synthesize and scavenge thiamine (17). In addition to different modes of acquisition for these nutrients, hypoxanthine and thiamine represent distinct metabolite classes: a nucleoside important for nucleic acid synthesis and a cofactor for apicoplast and mitochondrial metabolism, respectively.

Our approach to nutrient deprivation by limiting hypoxanthine or thiamine is similar to other studies that induced nutrient stress by reduction of a single amino acid (33, 34) or select metabolite classes (22, 35, 36) (Fig. 6A). However, we also observed superior recovery when parasites were grown in HPLM, a physiological medium formulation that has reduced concentrations of multiple nutrients (13). To our knowledge, this is the first use of HPLM as a media base for the propagation of P. falciparum; to induce starvation in previous studies, nutrient-rich RPMI-based media were diluted (36), producing a blanket reduction in nutrient levels. Regardless of the approach, the P. falciparum response to artemisinin treatment, when not protected by this supraphysiological glut of nutrients, had not been previously evaluated. This study begins to connect disparate observations to improve our understanding of how the environment impacts cellular responses.

### Molecular connections between nutrient limitation and DHA survival.

We demonstrate that metabolic priming triggers broad alterations of translational regulation (Fig. 5C and 6B), including genes associated with the UPR and proteasomal degradation. Previous studies in *Plasmodium* connected nutrient limitation with translational regulation (via eIF2α phosphorylation [33, 35]) and separately, eIF2α phosphorylation with survival of DHA treatment (37) (Fig. 6A and C). Our study establishes a direct link between nutrient limitation and DHA survival (Fig. 6B).

In higher eukaryotes, phosphorylation of eIF2α can promote activation of the PI3K complex and increase transcription of autophagy-related genes (38, 39). In *Plasmodium*, increased PI3K activity mediates artemisinin resistance (40). Further, polymorphisms in autophagy-related genes, such as ATG18 and ATG7, are associated with decreased artemisinin sensitivity in Southeast Asian parasite populations (21, 41). Recent studies support the role of eIF2α phosphorylation in promoting autophagy in *Plasmodium* (22). Despite growing evidence connecting translational regulation and autophagy in the parasite, we did not observe evidence that autophagy plays a prominent role in the protective effects of metabolic priming (Fig. S5 and Fig. 5C). This finding indicates that there is more to learn about intrinsic stress responses in *Plasmodium* parasites.

A surprising outcome of our study was the discovery that nutrient limitation triggered many responses that mimic an artemisinin tolerance-like transcriptional state (Fig. 6B and C). This adaptive response, which was stimulated by all priming conditions (Fig. 5), shifted the expression toward genes that had previously been linked to genetic artemisinin tolerance (Table 2). Future investigations should determine the longevity of priming-induced prosurvival signals and whether this adaptation facilitates development of genetic tolerance. This phenomenon has been observed in the development of antibiotic-resistant bacteria (42) and was recently proposed for clinical *Plasmodium* artemisinin resistance (26). Of note, more than half of the top transcriptional markers for resistance identified by Zhu et al. (26) were significantly altered (by adjusted *P* value) in the same direction in at least one of our priming conditions (32/58 genes).

### Translation from *in vitro* to *in vivo* results.

Analyses of how the *in vitro* environment affects pathogens outside their native environment remains limited. Alterations of metabolism that influence pathogen drug sensitivity can lead to discordance between *in vitro* results and *in vivo* responses. Our data emphasize the importance of the nutritional environment, whether standard *in vitro* culture conditions or more *in vivo*-like environments, on malaria parasite survival (Fig. 6B). Moving forward, the use of physiological HPLM-based media may be appropriate for select assessments where clinical translation is paramount. Future studies should also investigate if the nutritional environment is relevant to other antimalarial drugs and how prosurvival pathways can be targeted to enhance their killing potential.

### Relevance for the *in vivo* host environment.

Increasing resistance to artemisinin and its partner drugs necessitates development of new antimalarial drugs. Nucleotide metabolism is a potential future drug target in P. falciparum; antimalarials are currently being developed to inhibit both purine scavenging (including hypoxanthine) and pyrimidine biosynthesis (43 to 46). As some of these candidates move closer to use in the clinic, it is imperative to understand how parasites respond to the stress of nucleotide deprivation. Similarly, thiamine limitation has physiological relevance to P. falciparum infection as clinical thiamine deficiency is common in malaria regions of endemicity, such as Southeast Asia (47, 48), and evidence suggests malaria infection exacerbates this deficiency (49). Additionally, metabolic network reconstructions imply that drug resistance status can alter metabolism, making parasites more reliant on thiamine import for survival (50). This metabolic shift is corroborated by increased expression in artemisinin-tolerant isolates of thiamine pyrophosphokinase, a gene necessary for conversion of imported thiamine to the active thiamine pyrophosphate form (51).

Combining artemisinin with partner drugs of distinct mechanisms of action has contributed to the effectiveness of antimalarial drugs over recent decades. Such combination therapies minimize the development of tolerance or resistance. Yet, the potential for partner drugs that target metabolism (as described above) to induce an artemisinin tolerant-like transcriptional state poses an unexpected challenge that should be considered in the development of new antimalarial combinations.

Combating antimalarial drug resistance is especially critical as the spread of genetic artemisinin tolerance threatens to undo progress in reducing malaria mortality (52). However, the challenges of fighting drug resistance are not unique to malaria. The interplay among nutrient limitation, prosurvival pathways, and sensitivity to drug treatment (Fig. 6) has important implications for the evolution of drug resistance across diverse pathogens. How microbes adjust their metabolism in ways that impact survival of current and future drugs is an important avenue of research to prevent and slow the spread of drug resistant pathogens.

## MATERIALS AND METHODS

### Parasites and growth.

Plasmodium falciparum lines MRA 150 (Dd2), MRA 1000 (NF54), and MRA 1238 were obtained from the Malaria Research and Reference Reagent Resource Center (MR4, BEI Resources). The ATG8 conditional TetR-Dozi knockdown line was a generous gift from Ellen Yeh (Stanford University, Stanford, CA). *Plasmodium* cultures were maintained in A+ human erythrocytes (Valley Biomedical, Winchester, VA) at 3% hematocrit in RPMI 1640 HEPES (Sigma-Aldrich, St. Louis, MO) supplemented with 0.5% Albumax II Lipid-Rich BSA (ALB, Sigma-Aldrich, St. Louis, MO) and 50 mg/L hypoxanthine (Thermo Fisher Scientific, Waltham, MA). This formulation is referred to here as “standard media.” ATG8 TetR-DOZI parasites were maintained with 0.5 μM anhydrotetracycline (Sigma-Aldrich, St. Louis, MO) or supplemented with isopentyl pyrophosphate (Isoprenoids, LC, Tampa, FL), as previously described (20). Cultures were grown at 37°C and individually flushed with 5% oxygen, 5% carbon dioxide, and 90% nitrogen gas. Dilution of cultures with uninfected erythrocytes and changing of culture medium were performed every other day. Parasitemia was determined by flow cytometry using SYBR Green I staining and kept below 2% during maintenance. Cultures were confirmed negative for mycoplasma approximately monthly using a LookOut Mycoplasma PCR detection kit (Sigma-Aldrich, St. Louis, MO).

### Metabolic priming.

Low hypoxanthine medium was made using RPMI 1640 HEPES base with reduced exogenous hypoxanthine. A 2-mM hypoxanthine stock solution prepared in DMSO was diluted 1:4,000 to media to a final concentration of 0.5 μM. Thiamine-free medium was custom ordered to be identical to RPMI 1640 HEPES without the addition of thiamine hydrochloride. Hypoxanthine was added to thiamine-free media to 50 mg/L. Human Plasma-Like Media (HPLM, Thermo Fisher Scientific, Waltham, MA) was used without added HEPES or exogenous hypoxanthine. All media formulations were supplemented with 0.5% Albumax II Lipid-Rich BSA.

Uninfected erythrocytes were preincubated prior to use in priming experiments in the appropriate low-nutrient medium for 48 h at 37°C, 3 to 6% hematocrit, and 5% oxygen, 5% carbon dioxide, and 90% nitrogen gas. Preincubated uninfected erythrocytes were seeded with infected erythrocyte culture to a starting parasitemia of <0.5% and resuspended in control medium for nonprimed samples or low-nutrient medium for primed samples. Cultures were allowed to incubate for the prescribed period of time dependent on the condition (Table 1). For 72-h priming conditions, medium was refreshed at 48 h. For HPLM conditions, parasites were adapted to HPLM-based media for at least 7 days prior to drug treatment, and medium was refreshed every 48 h.

Aliquots were taken for flow cytometry measurement with SYBR Green I and MitoProbe DiIC1(5) kit (both Thermo Fisher Scientific, Waltham, MA). Primed samples were compared to nonprimed counterparts for reduction in growth, mitochondrial membrane potential (MMP), and percentage of ring stage parasites. Priming was considered successful when the following conditions were met for low-hypoxanthine primed samples compared to nonprimed: (i) an approximately >10% growth decrease, (ii) little to no decrease in MMP (approximately <10% drop), and (iii) ring stage percentages within approximately 10% of nonprimed. Priming in thiamine-free and HPLM conditions was considered successful if both of the first two conditions were met.

Following successful priming, both primed and nonprimed samples were treated with either 200 nM dihydroartemisinin (DHA; Sigma-Aldrich, St. Louis, MO) or DMSO for 6 h. DHA is an artemisinin derivative that causes widespread damage within parasites by promiscuously alkylating different classes of biomolecules, including heme and numerous proteins, thereby rapidly disrupting multiple biological pathways (53 to 55). Two hundred nM DHA was selected as this concentration is sufficient to cause a high level of parasite killing while still allowing for exponential regrowth to be measured within our experimental time frame (<14 days). To remove drug, cultures were washed three times in media. Low-hypoxanthine and thiamine-free samples were resuspended in normal, full-nutrient RPMI + Albumax + hypoxanthine medium and returned to the incubator. For HPLM conditions, parasites were maintained in HPLM during post-DHA recovery. Cultures were measured every other day with SYBR Green I and MitoProbe for 10 to14 days post drug treatment.

Biological replicates were performed in technical duplicate. For statistical analysis, technical duplicates were averaged into one value per biological replicate. Growth data sets 10 days post-DHA were then log transformed and tested for normality by Shapiro-Wilks test prior to running a repeated-measures two-way ANOVA followed by Sidak’s multiple-comparison test. RSA and TSA were also tested for normality by Shapiro–Wilk test then analyzed by ordinary two-way ANOVA followed by Dunnett’s multiple-comparison test. Analysis and visualizations were made using GraphPad Prism v. 7.04.

### Ring and trophozoite stage survival assays.

Survival assays were performed as previously described with minor modifications (56). Briefly, all metabolically primed samples were suspended in the relevant media at 36 h, 72 h, or ≥7 days prior to drug treatment for thiamine-, hypoxanthine-, and HPLM-primed conditions, respectively. Approximately 30 h prior to 0 to 3 h ring generation, 35-mL cultures were synchronized with 5% d-sorbitol and allowed to progress until the culture was predominantly schizonts. Schizonts were isolated by layering 4 mL of culture over 4 mL of 75% Percoll, centrifugation, and collection of the intermediate band. Isolated schizonts were washed then added to uninfected erythrocytes in the appropriate media formulation. Exactly 3 h later, a rapid d-sorbitol synchronization was performed (10 min at 37°C followed by 5 s vortex) to remove any uninvaded late-stage parasites. Parasites for TSAs were returned to the incubator for exactly 24 h before drug treatment, whereas parasites for RSAs were immediately pulsed with either DMSO or DHA (200 nM or 700 nM) for 6 h. Following drug treatment, all cells were washed multiple times and returned to RPMI or HPLM + ALB media for 66 h prior to assessment of survival by flow cytometry measurement with SYBR Green I and MitoProbe DiIC1(5) kit.

### Immunoblotting.

Anti-ATG8 antibody was a gift from Ellen Yeh (Stanford University, Stanford, CA). Anti-ATG8 was used in conjunction with goat anti-guinea pig IgG H&L (Alexa Fluor 488) (Abcam, Waltham, MA). Bound antibodies were detected on a Bio-Rad Imager and quantified using Image Lab Software (Bio-Rad, Hercules, CA).

### RNAseq and analysis.

Ring stage samples were generated by synchronization with 5% sorbitol 88 h and 44 h prior to harvest to accommodate the ~44-h erythrocytic cycle duration of Dd2 (57). Primed sample groups were grown as described above in their designated media for 72 h, 36 h, and 7 days prior to extraction for hypoxanthine, thiamine, and HPLM groups, respectively. Starting 24 h prior to RNA extraction, blood smears were assessed every 3 h until reinvasion was observed to approximate the age of rings in final samples. In all RNAseq samples, reinvasion was first observed 18 h prior to harvest. At sample collection, erythrocytes were lysed in 0.15% saponin before RNA was extracted from parasite pellets using a Direct-zol RNA Miniprep kit (Zymo Research) according to the manufacturer’s instructions. An aliquot of each sample was taken for assessment with a 2100 Bioanalyzer using the RNA 6000 Pico assay (Agilent Technologies, USA) before the remainder of the sample was snap-frozen in liquid nitrogen and stored at –80C.

Samples were shipped to Genewiz, Inc. for standard RNAseq with polyA selection. Raw reads obtained from Genewiz, Inc. were checked for overall quality and trimmed to remove adapters and low-quality bases from 3′ ends (with parameter -q 25) using TRIMGALORE (v. 0.6.7). STAR (v. 2.7.9a) was used to align trimmed reads with parameters –genomeSAindexNbases 11 and –alignIntronMax 500. Featurecounts (v. 2.0.1) was used for gene counting with parameter -t “exon.” Differential expression analysis was done using DESeq2 (v. 1.34.0). Genes were considered significantly differentially expressed (DEGs) if the following criteria were met: (i) Benjamini-Hochberg adjusted *P* value of ≤0.05 and (ii) log_2_-fold change of ≥1.0 or ≤−1.0. DESeq2 normalized read counts of DEGs presented in Table 2 were used for generation of a hierarchical clustering heatmap of Pearson’s correlation coefficients.

Parasite staging was estimated using the CIBERSORTx online platform. CIBERSORTx was used to generate a signature matrix of gene expression profiles from publicly available 10× genomics single-cell P. falciparum RNAseq data (58, 59). Data from Howick et al. contained raw count data for 6,737 P. falciparum intraerythrocytic cells categorized into ring, early trophozoite, late trophozoite, and schizont stages (59). Staging proportions from our bulk RNAseq samples were estimated using previously described parameters (60). Visualizations and analyses were generated in R version 4.1.2 using Tidyverse (v. 1.3.1) packages.

Gene set enrichment analysis (GSEA) was performed using fgsea (v. 1.20.0). In order to construct the “ART Tolerance” gene set for GSEA analysis (Table S3), we identified genes that were associated with decreased artemisinin sensitivity from 6 studies (27 to 32). We only considered samples that were under basal (nontreated) conditions, and where relevant, we used study-specific significance metrics. For Witkowski et al., we included genes that were differentially expressed in drug-selected artemisinin-tolerant parasite lines. For Siddiqui et al., we included all proteins with differential abundance in artemisinin-tolerant parasite lines. For Demas et al., we included all genes with mutations from drug-selected artemisinin-tolerant parasite lines. For Rocamora et al., we included all genes with mutations from drug-selected artemisinin-resistant parasite lines as well as genes with transcripts that were differentially expressed in artemisinin-tolerant parasite lines. For Mok et al., we included genes that were differentially expressed in isogenic artemisinin-tolerant parasite lines at schizont/early ring stage. For Simmons et al., we included genes that were differentially expressed in an artemisinin-tolerant transposon insertion mutant parasite line at ring/early trophozoite parasite stages (6 h, 12 h, and 24 h [32]). 3D7 genome sequence for alignment and annotation, gene product descriptions, and metabolic pathway information was pulled from PlasmoDB (release 55). Visualizations and analyses were generated in R version 4.1.2 using Tidyverse (v. 1.3.1) packages.

### Data availability.

Raw RNA sequencing reads are available through NCBI BioProject (accession: PRJNA930026). All other raw data will be shared upon request.
