# Supplementary material for: Nutrient Limitation Mimics Artemisinin Tolerance in Malaria
Source: mBio. 2023 Apr 25;14(3):e00705-23. doi: 10.1128/mbio.00705-23 (PMC10294616; doi:10.1128/mbio.00705-23)
Supplement: TABLE S1 [file mbio.00705-23-s0001.pdf]

Supplemental Table 1. Data for all recovery post-DHA or post-vehicle control experiments. Each row represents one independent experiment. Asterisk (*) indicates experiment did not meet criteria for successful priming (**Supplementary Fig. 3**). Explanation of column headers are as follows: Line = parasite line used for the experiment. Priming stress = condition of nutrient priming used in experiment alongside non-primed control. Drug stress= indicates if the experiment used a DHA pulse (DHA) following priming or a vehicle control pulse (No Drug). Non-primed rings = the fraction of parasites within the non-primed control sample identified as rings by SYBR Green based flow cytometry. Primed rings = the fraction of parasites within the nutrient primed sample identified as rings by SYBR Green based flow cytometry. Priming growth reduction = parasitemia of both non-primed and primed samples were measured at the beginning and end of the priming period. Reduction in growth of the primed sample relative to the non-primed control is presented as a percentage. Priming MMP change = mitochondrial membrane potential (MMP) was measured at the conclusion of the priming period. Reduction in MMP of the primed sample relative to the non-primed control is presented as a percentage. Blood age at start = age of red blood cells at the start of each experiment presented as days post phlebotomy.

LINE	PRIMING STRESS	DRUG STRESS	D10% DIFFERENCE	NON-PRIMED %RINGS	PRIMED %RINGS	PRIMING GROWTH REDUCTION	PRIMING MMP CHANGE	BLOOD AGE AT START (DAYS)
**DD2**	Hypoxanthine	DHA	200	53.5	59	30	n.d.	14
**DD2**	Hypoxanthine	DHA	99	58.5	53.5	42	-3	24
**DD2 (RNASEQ)**	Hypoxanthine	DHA	172	95	93	48	3	13
**DD2**	Thiamine Free	DHA	104	72.5	68.5	-69	-18	14
**DD2**	Thiamine Free	DHA	137	56.5	57	-1	1	18
**DD2 (RNASeq)**	Thiamine Free	DHA	138	95	96	-1	1	14
**DD2**	HPLM	DHA	676	73.5	74.5	-4	-7	28
**DD2**	HPLM	DHA	151	76	78	0	-1	16
**NF54**	Hypoxanthine	DHA	20	47.5	45.5	14	-3	27
**NF54 (ATG8 ON)**	Hypoxanthine	DHA	304	61	54	60	2	24
**NF54 (ATG8 ON)**	Hypoxanthine	DHA	77	56.5	64	19	14	25
**NF54 (ATG8 OFF)**	Hypoxanthine	DHA	85	67	61	36	2	24
**NF54 (ATG8 OFF)**	Hypoxanthine	DHA	149	56.5	60.5	16	3	25
**MRA1238 (K13 MT)**	Hypoxanthine	DHA	142	66.5	63	36	n.d.	9
**NF54**	Thiamine Free	DHA	195	68.5	67.5	10	1	18
**NF54**	Thiamine Free	DHA	257	44.5	47	17	-2	18
**NF54**	Hypoxanthine	No Drug	-30	47.5	45.5	14	-3	27
**NF54**	Hypoxanthine	No Drug	-27	68.5	75	45	0	9
**DD2**	Hypoxanthine	No Drug	-38	60	56	24	-1	12
**NF54**	Hypoxanthine	No Drug	-9	60.5	58.5	37	-3	27
**DD2**	Thiamine Free	No Drug	-5	48.5	44.5	7	-2	16

**DD2**	Thiamine Free	No Drug	29	63	67	-2	3.5	14
**DD2**	HPLM	No Drug	111	76	76	-14	n.d.	21
**DD2**	HPLM	No Drug	-71	76	78	0	-1	16
***NF54 (ATG8 ON)**	Hypoxanthine	DHA	-20	72	68	9	0	16
***D6**	Hypoxanthine	DHA	-8	62	32	66	1	20
