# Supplementary material for: Nutrient Limitation Mimics Artemisinin Tolerance in Malaria
Source: mBio. 2023 Apr 25;14(3):e00705-23. doi: 10.1128/mbio.00705-23 (PMC10294616; doi:10.1128/mbio.00705-23)
Supplement: FIG S1 [file mbio.00705-23-s0004.pdf]

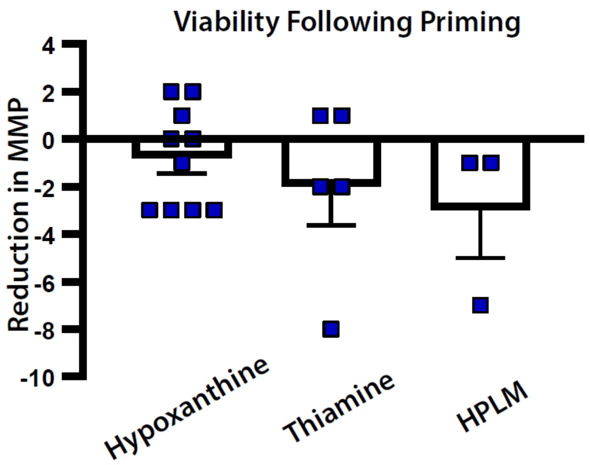


**Supplemental Figure 1. Metabolic priming does not drastically impact parasite viability.** Successful low nutrient metabolic priming leads to very small decreases in viability compared to standard media, non-primed controls. The percentage of parasites (SYBR Green I cells) also positive for MitoProbe DiIC1(5) staining (an indicator of mitochondrial membrane potential; MMP) was used as a proxy for determining viability. *N*=3-10 per condition. Bars represent S.E.M.
