# Supplementary material for: Nutrient Limitation Mimics Artemisinin Tolerance in Malaria
Source: mBio. 2023 Apr 25;14(3):e00705-23. doi: 10.1128/mbio.00705-23 (PMC10294616; doi:10.1128/mbio.00705-23)
Supplement: FIG S2 [file mbio.00705-23-s0005.pdf]

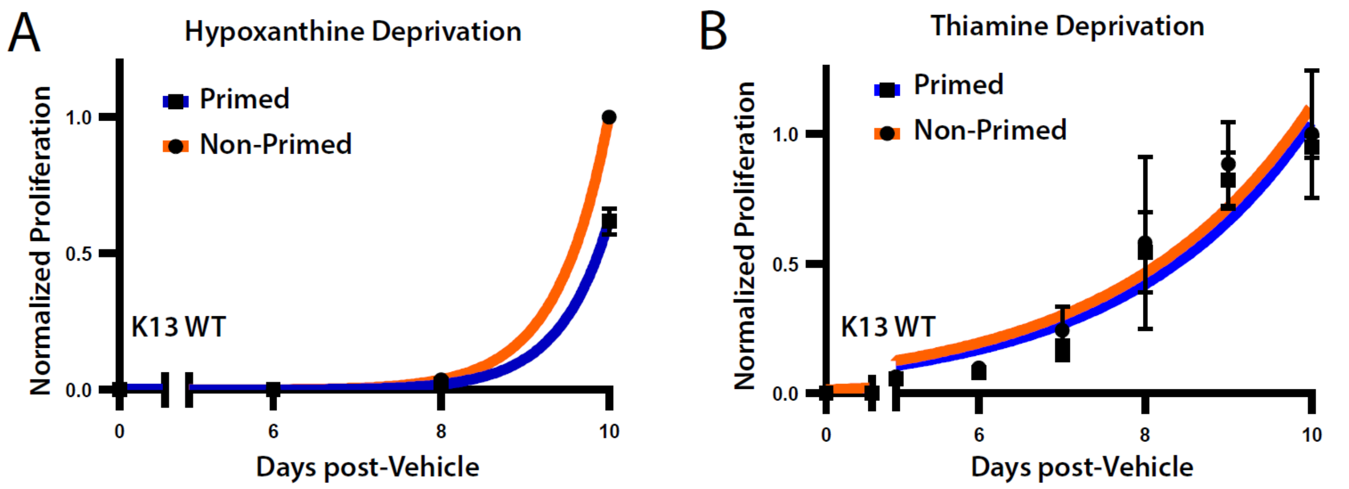

Supplemental Figure 2. Metabolic priming does not impact post-DHA recovery without drug pulse. A-B) Growth in standard media following metabolic priming under A) low hypoxanthine or B) thiamine-free conditions (Table 1) without a DHA drug pulse (vehicle is DMSO). Bars represent S.E.M. of technical replicates within one representative experiment. Results from independent assays are detailed in Supplemental Table 1 (*N*=2 per condition).
