# Supplementary material for: Nutrient Limitation Mimics Artemisinin Tolerance in Malaria
Source: mBio. 2023 Apr 25;14(3):e00705-23. doi: 10.1128/mbio.00705-23 (PMC10294616; doi:10.1128/mbio.00705-23)
Supplement: FIG S3 [file mbio.00705-23-s0006.pdf]

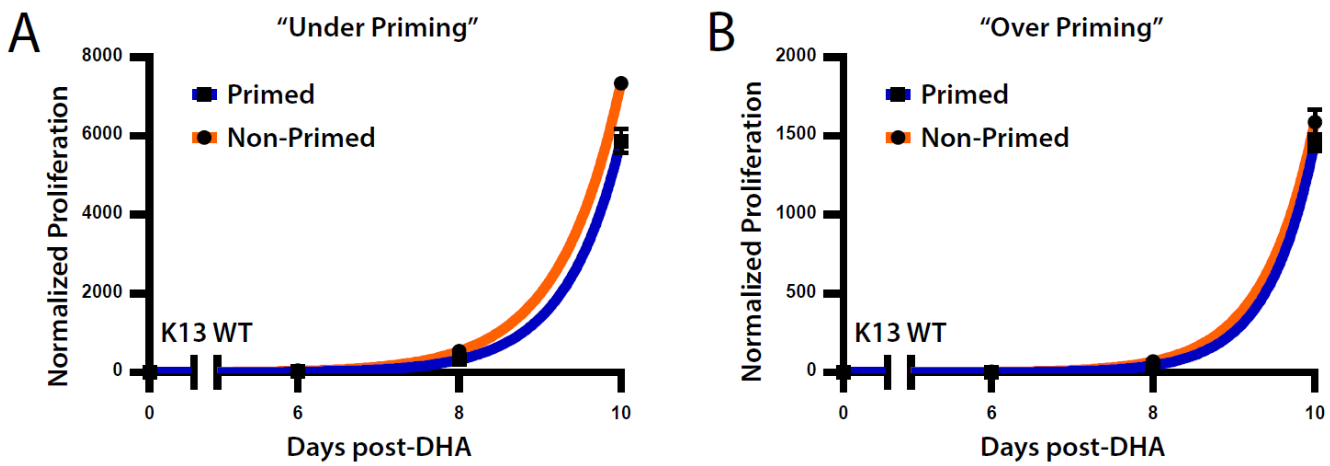

Supplemental Figure 3. Increased post-DHA recovery requires a specific range of stress during metabolic priming. A-B) Post-DHA recovery in standard media following low hypoxanthine metabolic priming where priming led to A) less than 10% growth reduction or B) greater than ~60% growth reduction and >10% reduction in MMP compared to non-primed controls. Bars represent S.E.M. of technical replicates within one independent experiment.
