# Supplementary material for: Nutrient Limitation Mimics Artemisinin Tolerance in Malaria
Source: mBio. 2023 Apr 25;14(3):e00705-23. doi: 10.1128/mbio.00705-23 (PMC10294616; doi:10.1128/mbio.00705-23)
Supplement: FIG S4 [file mbio.00705-23-s0007.pdf]

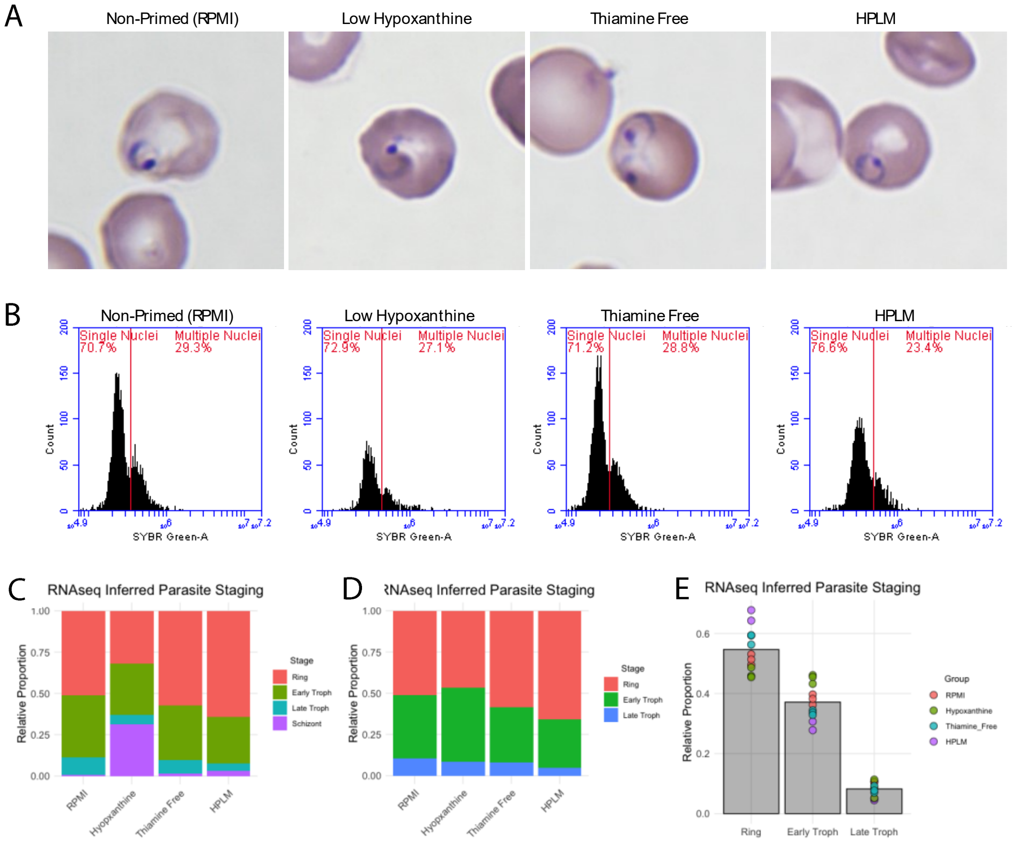

Supplemental Figure 4. Priming does not affect parasite stage makeup. A) Micrographs of synchronous ring stage parasites after priming (Magnification = 100x). B) Flow cytometry plots of synchronous non-primed and primed infected red blood cells. Plots are partitioned into single nuclei (early-stage parasites) and multiple nuclei (late-stage parasites) determined by SYBR Green I DNA staining intensity. C-D) Distribution of parasite stages computationally inferred from RNA expression profiles before (C) and after (D) removal of the schizont/uninvaded merozoite fraction. E) Distribution of inferred staging from (D) visualized across all treatment groups.
