# Supplementary material for: Nutrient Limitation Mimics Artemisinin Tolerance in Malaria
Source: mBio. 2023 Apr 25;14(3):e00705-23. doi: 10.1128/mbio.00705-23 (PMC10294616; doi:10.1128/mbio.00705-23)
Supplement: FIG S5 [file mbio.00705-23-s0008.pdf]

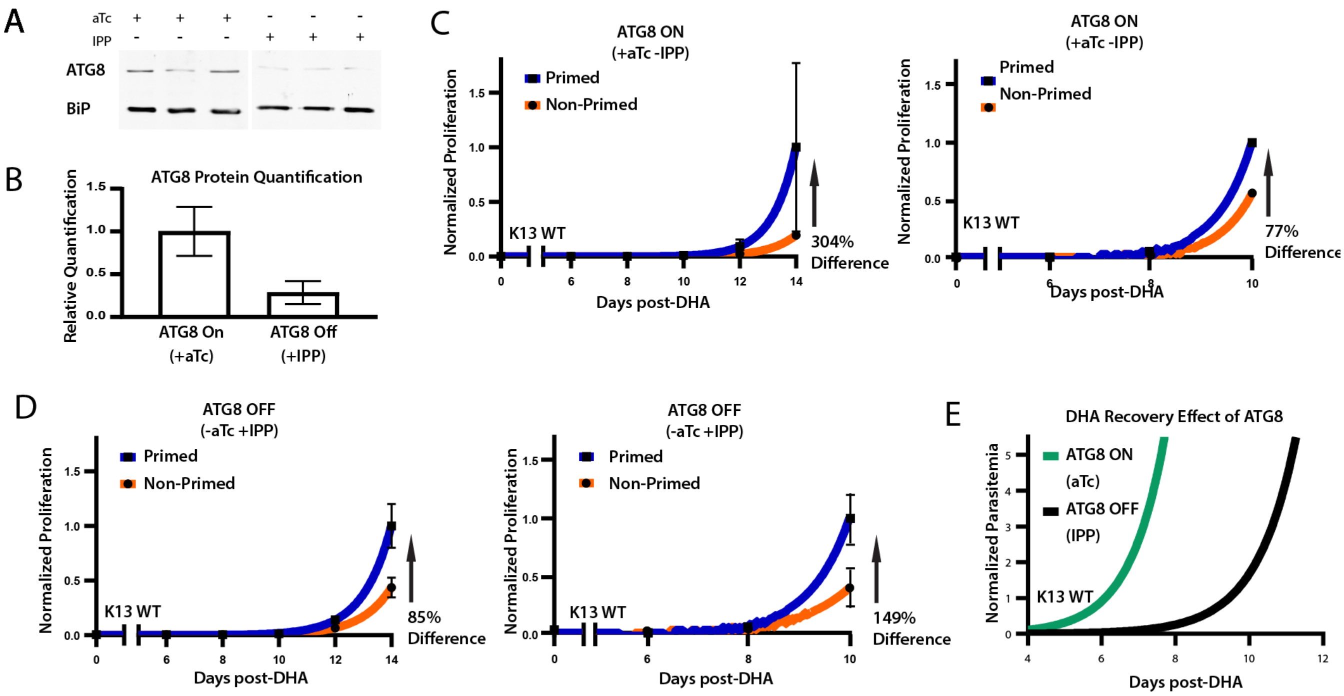

Supplemental Figure 5. Priming improves post-DHA survival regardless of autophagy status. A-B) Effect of knockdown on ATG8 protein levels in ATG8 TetR-Dozi parasites. Bars represent S.E.M. (*N*=3). C-D) Post-DHA recovery in two independent biological experiments of hypoxanthine primed ATG8 TetR-Dozi parasites with ATG8 expression on (C) or off (D). Bars represent S.E.M. of technical replicates within one independent experiment. E) Growth recovery of non-primed parasites differing in ATG8 expression status (*N*=2). aTc = anhydrotetracycline; small molecule that allows induction of ATG8 expression. IPP = isoprenoids; necessary metabolite for *P. falciparum* survival in the absence of ATG8.
