# Supplementary material for: Nutrient Limitation Mimics Artemisinin Tolerance in Malaria
Source: mBio. 2023 Apr 25;14(3):e00705-23. doi: 10.1128/mbio.00705-23 (PMC10294616; doi:10.1128/mbio.00705-23)
Supplement: TABLE S3 [file mbio.00705-23-s0003.pdf]

Supplemental Table 3. Studies measuring basal differences between artemisinin resistant and sensitive parasites using 'Omics approaches.

Publication	Level	No. Genes Used
Demas, et al. 2018 PNAS	Genome	7
Rocamora, et al. 2018 PloS Pathogens	Genome; Transcript	47
Mok, et al. 2021 Nature Comm.	Transcript	80
Siddiqui, et al. 2017 J. Infectious Disease	Protein	12
Witkowksi, et al. 2010 AAC.	Transcript	9
Simmons, et al. 2023 Sci Rep.	Transcript	32
	**Total**	**187**
	**Unique Genes**	**181**

Gene ID	Publication	Product Description
PF3D7_1454700	Rocamora, et al. 2018 PloS Path	6-phosphogluconate dehydrogenase, decarboxylating
PF3D7_1457200	Rocamora, et al. 2018 PloS Path	thioredoxin 1
PF3D7_1457000	Rocamora, et al. 2018 PloS Path	signal peptide peptidase
PF3D7_0704800	Rocamora, et al. 2018 PloS Path	protein phosphatase PPM12, putative
PF3D7_0730300	Rocamora, et al. 2018 PloS Path	AP2 domain transcription factor AP2-L, putative
PF3D7_1115700	Rocamora, et al. 2018 PloS Path	cysteine proteinase falcipain 2a
PF3D7_1141800	Rocamora, et al. 2018 PloS Path	EELM2 domain-containing protein, putative
PF3D7_1427100	Rocamora, et al. 2018 PloS Path	lipase, putative
PF3D7_0420300	Rocamora, et al. 2018 PloS Path	AP2 domain transcription factor, putative
PF3D7_0528300	Rocamora, et al. 2018 PloS Path	conserved protein, unknown function
PF3D7_0617100	Rocamora, et al. 2018 PloS Path	AP-2 complex subunit alpha, putative
PF3D7_0810600	Rocamora, et al. 2018 PloS Path	ATP-dependent RNA helicase DBP1, putative
PF3D7_1230000	Rocamora, et al. 2018 PloS Path	TBC domain-containing protein, putative
PF3D7_1368400	Rocamora, et al. 2018 PloS Path	ribosomal protein L1, putative
PF3D7_1478100	Rocamora, et al. 2018 PloS Path	Plasmodium exported protein (hyp13), unknown function
PF3D7_1200800	Rocamora, et al. 2018 PloS Path	serine/threonine protein kinase, FIKK family
PF3D7_0702300	Rocamora, et al. 2018 PloS Path	sporozoite threonine and asparagine-rich protein
PF3D7_1030100	Rocamora, et al. 2018 PloS Path	pre-mRNA-splicing factor ATP-dependent RNA helicase PRP22, putative

PF3D7_1300300	Rocamora, et al. 2018 PloS Path	erythrocyte membrane protein 1, PfEMP1
PF3D7_1479000	Rocamora, et al. 2018 PloS Path	acyl-CoA synthetase
PF3D7_0933500	Rocamora, et al. 2018 PloS Path	gamma-tubulin complex component, putative
PF3D7_0812500	Rocamora, et al. 2018 PloS Path	RNA-binding protein, putative
PF3D7_0113400	Rocamora, et al. 2018 PloS Path	Plasmodium exported protein, unknown function
PF3D7_1110400	Rocamora, et al. 2018 PloS Path	RNA-binding protein, putative
PF3D7_1116800	Rocamora, et al. 2018 PloS Path	heat shock protein 101
PF3D7_1235300	Rocamora, et al. 2018 PloS Path	CCR4-NOT transcription complex subunit 4, putative
PF3D7_1351000	Rocamora, et al. 2018 PloS Path	phosphatidylinositol transfer protein, putative
PF3D7_0609900	Rocamora, et al. 2018 PloS Path	tetratricopeptide repeat protein, putative
PF3D7_1406200	Rocamora, et al. 2018 PloS Path	transcription elongation factor SPT6, putative
PF3D7_0400400	Rocamora, et al. 2018 PloS Path	erythrocyte membrane protein 1, PfEMP1
PF3D7_0600200	Rocamora, et al. 2018 PloS Path	erythrocyte membrane protein 1, PfEMP1
PF3D7_1001200	Rocamora, et al. 2018 PloS Path	acyl-CoA binding protein, isoform 2, ACBP2
PF3D7_0400500	Rocamora, et al. 2018 PloS Path	rifin
PF3D7_0712900	Rocamora, et al. 2018 PloS Path	erythrocyte membrane protein 1, PfEMP1
PF3D7_1139100	Rocamora, et al. 2018 PloS Path	RNA-binding protein, putative
PF3D7_0201600	Rocamora, et al. 2018 PloS Path	PHISTb domain-containing RESA-like protein 1
PF3D7_0302300	Rocamora, et al. 2018 PloS Path	erythrocyte membrane protein 1 (PfEMP1), pseudogene
PF3D7_0600400	Rocamora, et al. 2018 PloS Path	erythrocyte membrane protein 1, PfEMP1
PF3D7_1208400	Rocamora, et al. 2018 PloS Path	amino acid transporter, putative
PF3D7_1470800	Rocamora, et al. 2018 PloS Path	conserved Plasmodium protein, unknown function
PF3D7_1245500	Rocamora, et al. 2018 PloS Path	conserved Plasmodium protein, unknown function
PF3D7_0409100	Rocamora, et al. 2018 PloS Path	U4/U6 small nuclear ribonucleoprotein PRP31, putative
PF3D7_0617400	Rocamora, et al. 2018 PloS Path	erythrocyte membrane protein 1, PfEMP1
PF3D7_0918300	Rocamora, et al. 2018 PloS Path	eukaryotic translation initiation factor 3 subunit F, putative

PF3D7_0906400	Rocamora, et al. 2018 PloS Path	dynein intermediate light chain, putative
PF3D7_0107900	Rocamora, et al. 2018 PloS Path	conserved Plasmodium protein, unknown function
PF3D7_0315000	Rocamora, et al. 2018 PloS Path	zinc finger protein, putative
PF3D7_0701900	Mok, et al. 2021 Nat Comm	Plasmodium exported protein, unknown function
PF3D7_1252600	Mok, et al. 2021 Nat Comm	esterase, putative
PF3D7_0702100	Mok, et al. 2021 Nat Comm	Plasmodium exported protein (PHISTb), unknown function, pseudogene
PF3D7_1341700	Mok, et al. 2021 Nat Comm	conserved Plasmodium protein, unknown function
PF3D7_1343700	Mok, et al. 2021 Nat Comm	kelch protein K13
PF3D7_1218500	Mok, et al. 2021 Nat Comm	dynamin-like protein, putative
PF3D7_1252900	Mok, et al. 2021 Nat Comm	Plasmodium exported protein, unknown function
PF3D7_0102200	Mok, et al. 2021 Nat Comm	ring-infected erythrocyte surface antigen
PF3D7_0401900	Mok, et al. 2021 Nat Comm	acyl-CoA synthetase
PF3D7_1352900	Mok, et al. 2021 Nat Comm	Plasmodium exported protein, unknown function
PF3D7_1002100	Mok, et al. 2021 Nat Comm	EMP1-trafficking protein
PF3D7_0933000	Mok, et al. 2021 Nat Comm	CSTF domain-containing protein, putative
PF3D7_1108000	Mok, et al. 2021 Nat Comm	IWS1-like protein, putative
PF3D7_1461600	Mok, et al. 2021 Nat Comm	splicing factor 3B subunit 2, putative
PF3D7_1006800	Mok, et al. 2021 Nat Comm	G-strand-binding protein 2
PF3D7_0612900	Mok, et al. 2021 Nat Comm	nucleolar GTP-binding protein 1, putative
PF3D7_0901900	Mok, et al. 2021 Nat Comm	probable protein, unknown function
PF3D7_1000800	Mok, et al. 2021 Nat Comm	stevor, pseudogene
PF3D7_1361900	Mok, et al. 2021 Nat Comm	proliferating cell nuclear antigen 1
PF3D7_0317200	Mok, et al. 2021 Nat Comm	cdc2-related protein kinase 4
PF3D7_1304100	Mok, et al. 2021 Nat Comm	DNA ligase I
PF3D7_1320100	Mok, et al. 2021 Nat Comm	ATP-dependent Clp protease adapter protein ClpS
PF3D7_0811600	Mok, et al. 2021 Nat Comm	conserved protein, unknown function
PF3D7_0315900	Mok, et al. 2021 Nat Comm	conserved Plasmodium protein, unknown function
PF3D7_1246200	Mok, et al. 2021 Nat Comm	actin I
PF3D7_0935800	Mok, et al. 2021 Nat Comm	cytoadherence linked asexual protein 9
PF3D7_1223100	Mok, et al. 2021 Nat Comm	cAMP-dependent protein kinase regulatory subunit
PF3D7_1252100	Mok, et al. 2021 Nat Comm	rhoptry neck protein 3
PF3D7_0104200	Mok, et al. 2021 Nat Comm	StAR-related lipid transfer protein
PF3D7_1128900	Mok, et al. 2021 Nat Comm	conserved protein, unknown function
PF3D7_1467900	Mok, et al. 2021 Nat Comm	rab GTPase activator, putative
PF3D7_1145200	Mok, et al. 2021 Nat Comm	serine/threonine protein kinase, putative

PF3D7_1335100	Mok, et al. 2021 Nat Comm	merozoite surface protein 7
PF3D7_0831600	Mok, et al. 2021 Nat Comm	cytoadherence linked asexual protein 8
PF3D7_0722200	Mok, et al. 2021 Nat Comm	rhoptry-associated leucine zipper-like protein 1
PF3D7_1140400	Mok, et al. 2021 Nat Comm	conserved Plasmodium protein, unknown function
PF3D7_1206300	Mok, et al. 2021 Nat Comm	conserved Plasmodium protein, unknown function
PF3D7_1452000	Mok, et al. 2021 Nat Comm	rhoptry neck protein 2
PF3D7_0810300	Mok, et al. 2021 Nat Comm	protein phosphatase PPM5, putative
PF3D7_0419700	Mok, et al. 2021 Nat Comm	apical merozoite protein
PF3D7_1021700	Mok, et al. 2021 Nat Comm	VPS13 domain-containing protein, putative
PF3D7_0628100	Mok, et al. 2021 Nat Comm	HECT domain-containing protein 1, putative
PF3D7_0905500	Mok, et al. 2021 Nat Comm	conserved protein, unknown function
PF3D7_0802600	Mok, et al. 2021 Nat Comm	adenylyl cyclase beta
PF3D7_1022500	Mok, et al. 2021 Nat Comm	citrate synthase, mitochondrial, putative
PF3D7_0817600	Mok, et al. 2021 Nat Comm	conserved protein, unknown function
PF3D7_1035300	Mok, et al. 2021 Nat Comm	glutamate-rich protein GLURP
PF3D7_1035900	Mok, et al. 2021 Nat Comm	merozoites-associated armadillo repeats protein
PF3D7_1104900	Mok, et al. 2021 Nat Comm	calcium/calmodulin-dependent protein kinase, putative
PF3D7_1028700	Mok, et al. 2021 Nat Comm	merozoite TRAP-like protein
PF3D7_0614700	Mok, et al. 2021 Nat Comm	F-box protein FBXO6, putative
PF3D7_1246400	Mok, et al. 2021 Nat Comm	myosin A-tail interacting protein
PF3D7_1229800	Mok, et al. 2021 Nat Comm	myosin J, putative
PF3D7_1344300	Mok, et al. 2021 Nat Comm	zinc finger protein, putative
PF3D7_0407900	Mok, et al. 2021 Nat Comm	AAA family ATPase, putative
PF3D7_1133400	Mok, et al. 2021 Nat Comm	apical membrane antigen 1
PF3D7_0822900	Mok, et al. 2021 Nat Comm	PhIL1-interacting candidate PIC2
PF3D7_0210600	Mok, et al. 2021 Nat Comm	protein CERLI1
PF3D7_1036000	Mok, et al. 2021 Nat Comm	merozoite surface protein 11
PF3D7_1468400	Mok, et al. 2021 Nat Comm	C3H1-type zinc finger protein CZIF1
PF3D7_1243700	Mok, et al. 2021 Nat Comm	ubiquitin-conjugating enzyme E2, putative
PF3D7_0308300	Mok, et al. 2021 Nat Comm	PhIL1-interacting candidate PIC4
PF3D7_1251200	Mok, et al. 2021 Nat Comm	coronin
PF3D7_0821400	Mok, et al. 2021 Nat Comm	conserved Plasmodium protein, unknown function
PF3D7_1310700	Mok, et al. 2021 Nat Comm	PhIL1-interacting candidate PIC5
PF3D7_0515400	Mok, et al. 2021 Nat Comm	conserved protein, unknown function
PF3D7_1036500	Mok, et al. 2021 Nat Comm	conserved Plasmodium protein, unknown function
PF3D7_0424100	Mok, et al. 2021 Nat Comm	reticulocyte binding protein homologue 5

PF3D7_1125700	Mok, et al. 2021 Nat Comm	kelch domain-containing protein, putative
PF3D7_0214600	Mok, et al. 2021 Nat Comm	serine/threonine protein kinase STK2, putative
PF3D7_0828800	Mok, et al. 2021 Nat Comm	GPI-anchored micronemal antigen
PF3D7_1227700	Mok, et al. 2021 Nat Comm	protein KIC2
PF3D7_0402300	Mok, et al. 2021 Nat Comm	reticulocyte binding protein homologue 1
PF3D7_1238800	Mok, et al. 2021 Nat Comm	acyl-CoA synthetase
PF3D7_0731500	Mok, et al. 2021 Nat Comm	erythrocyte binding antigen-175
PF3D7_0408000	Mok, et al. 2021 Nat Comm	conserved Plasmodium protein, unknown function
PF3D7_1230700	Mok, et al. 2021 Nat Comm	protein transport protein SEC13
PF3D7_1433500	Mok, et al. 2021 Nat Comm	DNA topoisomerase 2
PF3D7_0410000	Mok, et al. 2021 Nat Comm	erythrocyte vesicle protein 1
PF3D7_1240100	Mok, et al. 2021 Nat Comm	early transcribed membrane protein 12
PF3D7_1251200	Demas, et al. 2018 PNAS	coronin
PF3D7_1433800	Demas, et al. 2018 PNAS	conserved Plasmodium protein, unknown function
PF3D7_1126100	Demas, et al. 2018 PNAS	autophagy-related protein 7, putative
PF3D7_0209600	Demas, et al. 2018 PNAS	transporter, putative
PF3D7_1121900	Demas, et al. 2018 PNAS	3-phosphoinositide-dependent protein kinase 1
PF3D7_1324300	Demas, et al. 2018 PNAS	conserved Plasmodium membrane protein, unknown function
PF3D7_1422400	Demas, et al. 2018 PNAS	nucleolar RNA-associated protein, putative
PF3D7_1343700	Siddiqui, et al. 2017 JID	kelch protein K13
PF3D7_0500800	Siddiqui, et al. 2017 JID	mature parasite-infected erythrocyte surface antigen
PF3D7_1116700	Siddiqui, et al. 2017 JID	dipeptidyl aminopeptidase 1
PF3D7_0406200	Siddiqui, et al. 2017 JID	parasitophorous vacuole membrane protein S16
PF3D7_0702500	Siddiqui, et al. 2017 JID	Plasmodium exported protein, unknown function
PF3D7_1343700	Siddiqui, et al. 2017 JID	kelch protein K13
PF3D7_1364800	Siddiqui, et al. 2017 JID	DNA-directed RNA polymerases I, II, and III subunit RPABC1, putative
PF3D7_0209800	Siddiqui, et al. 2017 JID	ATP-dependent RNA helicase UAP56
PF3D7_1447000	Siddiqui, et al. 2017 JID	40S ribosomal protein S5
PF3D7_0204500	Siddiqui, et al. 2017 JID	aspartate transaminase
PF3D7_1206200	Siddiqui, et al. 2017 JID	eukaryotic translation initiation factor 3 subunit C, putative
PF3D7_0320700	Siddiqui, et al. 2017 JID	signal peptidase complex subunit 2
PF3D7_0202000	Wikowski, et al. 2010 AAC	knob-associated histidine-rich protein
PF3D7_1372500	Wikowski, et al. 2010 AAC	stevor, pseudogene
PF3D7_1372600	Wikowski, et al. 2010 AAC	rifin
PF3D7_0818900	Wikowski, et al. 2010 AAC	heat shock protein 70

PF3D7_1012400	Wikowski, et al. 2010 AAC	hypoxanthine-guanine phosphoribosyltransferase
PF3D7_0202100	Wikowski, et al. 2010 AAC	liver stage associated protein 2
PF3D7_0300900	Wikowski, et al. 2010 AAC	stevor
PF3D7_0301700	Wikowski, et al. 2010 AAC	EMP1 trafficking protein PTP7
PF3D7_0528400	Wikowski, et al. 2010 AAC	palmitoyltransferase DHHC7
PF3D7_0401900	Simmons, et al. 2023 Sci Rep	acyl-CoA synthetase
PF3D7_1147400	Simmons, et al. 2023 Sci Rep	COX2 transmembrane domain-containing protein, putative
PF3D7_1115000	Simmons, et al. 2023 Sci Rep	conserved protein, unknown function
PF3D7_1229100	Simmons, et al. 2023 Sci Rep	multidrug resistance-associated protein 2
PF3D7_1432700	Simmons, et al. 2023 Sci Rep	protein-L-isoaspartate(D-aspartate) O-methyltransferase, putative
PF3D7_1137000	Simmons, et al. 2023 Sci Rep	U2 spliceosomal RNA
PF3D7_0302300	Simmons, et al. 2023 Sci Rep	erythrocyte membrane protein 1 (PfEMP1), pseudogene
PF3D7_1101300	Simmons, et al. 2023 Sci Rep	rifin
PF3D7_1231800	Simmons, et al. 2023 Sci Rep	asparagine-rich protein, putative
PF3D7_0623300	Simmons, et al. 2023 Sci Rep	EGF-like membrane protein, putative
PF3D7_0112200	Simmons, et al. 2023 Sci Rep	multidrug resistance-associated protein 1
PF3D7_1329500	Simmons, et al. 2023 Sci Rep	conserved protein, unknown function
PF3D7_0522500	Simmons, et al. 2023 Sci Rep	50S ribosomal protein L17, apicoplast, putative
PF3D7_1127100	Simmons, et al. 2023 Sci Rep	deoxyuridine 5'-triphosphate nucleotidohydrolase
PF3D7_0422300	Simmons, et al. 2023 Sci Rep	alpha tubulin 2
PF3D7_1038400	Simmons, et al. 2023 Sci Rep	gametocyte-specific protein
PF3D7_1331300	Simmons, et al. 2023 Sci Rep	signal peptidase complex catalytic subunit SEC11
PF3D7_0613400	Simmons, et al. 2023 Sci Rep	50S ribosomal protein L18, apicoplast, putative
PF3D7_0505800	Simmons, et al. 2023 Sci Rep	small ubiquitin-related modifier
PF3D7_0610400	Simmons, et al. 2023 Sci Rep	histone H3
PF3D7_1142700	Simmons, et al. 2023 Sci Rep	methyltransferase, putative
PF3D7_0624500	Simmons, et al. 2023 Sci Rep	anaphase-promoting complex subunit 11, putative
PF3D7_0411000	Simmons, et al. 2023 Sci Rep	AP2 domain transcription factor AP2-Z, putative
PF3D7_0312000	Simmons, et al. 2023 Sci Rep	HTH domain-containing protein, putative
PF3D7_1434800	Simmons, et al. 2023 Sci Rep	mitochondrial acidic protein MAM33, putative
PF3D7_0907900	Simmons, et al. 2023 Sci Rep	peptide deformylase
PF3D7_1119900	Simmons, et al. 2023 Sci Rep	protein transport protein SEC16, putative
PF3D7_1446600	Simmons, et al. 2023 Sci Rep	centrin-2
PF3D7_1477000	Simmons, et al. 2023 Sci Rep	Plasmodium exported protein (hyp17), unknown function, pseudogene
PF3D7_1320100	Simmons, et al. 2023 Sci Rep	ATP-dependent Clp protease adapter protein ClpS

PF3D7_0525700	Simmons, et al. 2023 Sci Rep	conserved protein, unknown function
PF3D7_1372200	Simmons, et al. 2023 Sci Rep	histidine-rich protein III
