# Supplementary material for: Nutrient Limitation Mimics Artemisinin Tolerance in Malaria
Source: mBio. 2023 Apr 25;14(3):e00705-23. doi: 10.1128/mbio.00705-23 (PMC10294616; doi:10.1128/mbio.00705-23)
Supplement: FIG S6 [file mbio.00705-23-s0009.pdf]

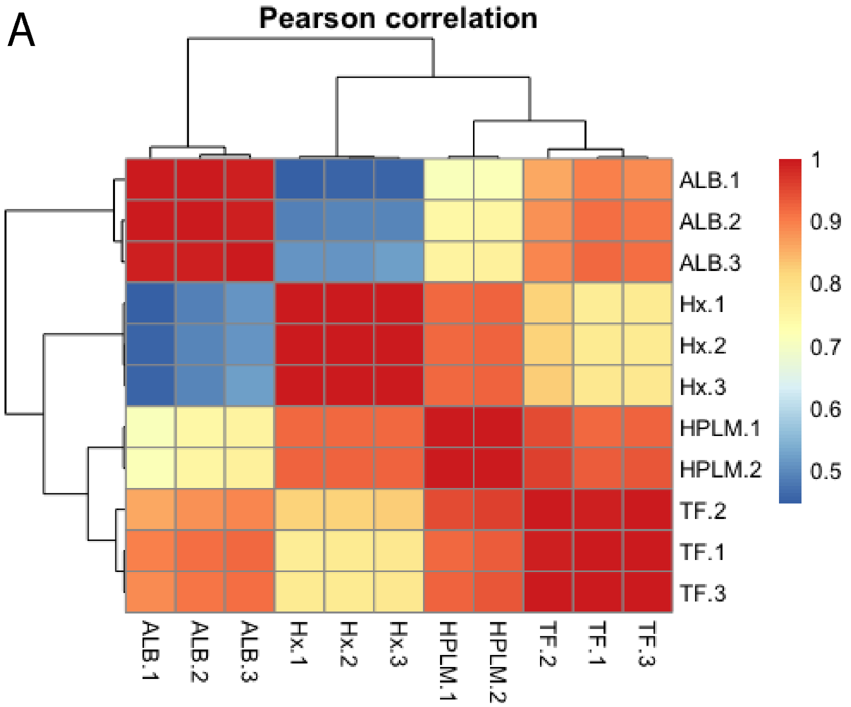

Supplemental Figure 6. Clustering on DEGs reveals differences between non-primed and treatment group samples but with low intergroup variability. Hierarchical clustering of Pearson's correlation coefficients utilizing counts of genes listed in Table 2. ALB = non-primed. Hx = hypoxanthine primed. HPLM = human plasma like media, TF = thiamine-free primed.
